# FAIR data retrieval for sensitive clinical research data in Galaxy

**DOI:** 10.1093/gigascience/giad099

**Published:** 2024-01-27

**Authors:** Jasper Ouwerkerk, Helena Rasche, John D Spalding, Saskia Hiltemann, Andrew P Stubbs

**Affiliations:** Clinical Bioinformatics Group, Department of Pathology, Erasmus Medical Center, 3015 CN, Rotterdam, the Netherlands; Clinical Bioinformatics Group, Department of Pathology, Erasmus Medical Center, 3015 CN, Rotterdam, the Netherlands; CSC–IT Center for Science, 02101 Espoo, Finland; Clinical Bioinformatics Group, Department of Pathology, Erasmus Medical Center, 3015 CN, Rotterdam, the Netherlands; Clinical Bioinformatics Group, Department of Pathology, Erasmus Medical Center, 3015 CN, Rotterdam, the Netherlands

**Keywords:** B1MG, FAIR, Galaxy, trio analysis

## Abstract

**Background:**

In clinical research, data have to be accessible and reproducible, but the generated data are becoming larger and analysis complex. Here we propose a platform for Findable, Accessible, Interoperable, and Reusable (FAIR) data access and creating reproducible findings. Standardized access to a major genomic repository, the European Genome-Phenome Archive (EGA), has been achieved with API services like PyEGA3. We aim to provide a FAIR data analysis service in Galaxy by retrieving genomic data from the EGA and provide a generalized “omics” platform for FAIR data analysis.

**Results:**

To demonstrate this, we implemented an end-to-end Galaxy workflow to replicate the findings from an RD-Connect synthetic dataset Beyond the 1 Million Genomes (synB1MG) available from the EGA. We developed the PyEGA3 connector within Galaxy to easily download multiple datasets from the EGA. We added the gene.iobio tool, a diagnostic environment for precision genomics, to Galaxy and demonstrate that it provides a more dynamic and interpretable view for trio analysis results. We developed a Galaxy trio analysis workflow to determine the pathogenic variants from the synB1MG trios using the GEMINI and gene.iobio tool. The complete workflow is available at WorkflowHub, and an associated tutorial was created in the Galaxy Training Network, which helps researchers unfamiliar with Galaxy to run the workflow.

**Conclusions:**

We showed the feasibility of reusing data from the EGA in Galaxy via PyEGA3 and validated the workflow by rediscovering spiked-in variants in synthetic data. Finally, we improved existing tools in Galaxy and created a workflow for trio analysis to demonstrate the value of FAIR genomics analysis in Galaxy.

Key Points

- Secure access to GA4GH EGA service using PyEGA3 Galaxy service
- Standard analysis for B1MG synthetic data
- Interactive gene variant detection for trio analysis with gene.iobio in Galaxy
- Created a tutorial associated with the Galaxy Training Network

## Findings

### Background

In the past few years, there have been many developments in Findable, Accessible, Interoperable, and Reusable (FAIR) data [1]. FAIR data are data and corresponding metadata that are (i) findable by both machines and humans, (ii) accessible using a standard open protocol, (iii) interoperable so they can easily be processed and analyzed, and (iv) resuable so the data can be understood by anyone and make analyses reproducible [2]. FAIR data allow researchers to reanalyze data with new genetic analysis tools not yet available at the time of data publication. For example, in a study on fusion genes, 24 novel fusions in breast cancer were found with the introduction of a new tool [3].

However, for many biomedical analyses, researchers are required to have considerable knowledge on using analysis tools. Moreover, many tools require knowledge of Unix commands or Python coding [4–6]. This creates a barrier for clinical researchers who want to reanalyze data, reducing the adoption of implementing FAIR principles and reanalyses.

The Galaxy platform [7] supports researchers in adopting these complex computation tools for their FAIR data analysis. Galaxy is an online analysis platform with a plethora of tools to perform text/table processing, omics analysis, machine learning, image analysis, and more. All these tools are maintained and developed by a growing community. Using the tools does not require any programming skills, and they are easy to share with colleagues and other researchers. At the end of the analysis, the workflow of tools can be exported to reproduce the analysis [7]. These workflows can be made discoverable by uploading the workflow to WorkflowHub [8], a registry for describing, sharing, and publishing scientific computational workflows. In addition, Galaxy already has 300+ tutorials describing workflows on genome assembly, ecology, meta-genomics, variant analysis, and more [9]. This is beneficial to many researchers since many complex Unix-based tools are essential for biomedical research. An example of such an application is Circos [6], which is a complicated visualization tool for comparing whole genomes. This tool has been implemented within Galaxy, which makes it simple for any researcher to create Circos plots [10].

Even though Galaxy is a well-established platform for analysis, it still lacks applications for retrieving access-controlled data from large repositories like the EGA. The EGA controls the accessibility to datasets using Data Access Committees (DACs). Requestors can access data from the EGA by contacting the DAC for the dataset of interest. DACs are generally formed by the organization that collected the data and performed the analysis. This allows researchers to access datasets of interest and also manage the accessibility of their data at the EGA [11].

In this work, we implemented PyEGA3 [12], a tool that can access controlled data from the EGA, within Galaxy. Here, access to datasets is managed via the EGA. Our implementation of the PyEGA3 tool allows to filter datasets, available on the EGA, based on their metadata and scale up analysis. This will be showcased by validating our workflow for trio analysis on family trios from the Beyond 1 Million Genomes (B1MG) project [13]. In trio analysis, the differences in DNA between the maternal, paternal, and affected child (i.e., proband) are analyzed to detect causative variants causing a particular disease in the proband. To perform the trio analysis, we added gene.iobio, a standalone web-based tool, to Galaxy [14]. The complete workflow, including data retrieval with PyEGA3, is implemented within Galaxy and uploaded to WorkflowHub for discoverability. In addition, we wrote a tutorial to explain our workflow in detail, which is associated with the Galaxy Training Network (GTN) [15]. This study shows it is feasible to adopt end-to-end scalable FAIR analysis of clinical data and ultimately for any future analysis on data available at the EGA.

### Results

#### PyEGA3

PyEGA3 was implemented to retrieve access-controlled data from the EGA in Galaxy. Authentication of the user is done by password and username. This information is encrypted using a Vault abstraction [16] when configured by the Galaxy administrator. In addition, LS Login (Previously ELIXIR Authentication and Authorization Infrastructure [AAI]) tokens can be used for authentication, if set up by the user. The tokens are stored in the Galaxy database and temporarily valid (1 hour by default) to shorten the window of time for a potential attack. Currently, the process of authentication is initiated by linking one’s EGA account to their LS Login (possible via https://ega.ebi.ac.uk:8443/ega-openid-connect-server/ega-login; previously ELIXIR AAI) account. Next, the user logs in to Galaxy via LS Login, which attaches the user’s GA4GH passport and access and refresh tokens to the user’s account in Galaxy. The refresh token is used to regularly refresh their credentials, allowing the Galaxy server to act on their behalf when the user requests it via tool execution. Upon executing a tool, assuming the tool is written to support it, the access token or, possibly in the future, passports are attached to the tool’s execution such that they can be used to authenticate the user. While currently the access token is implemented on an *ad hoc* basis, we intend to directly implement support for this type of tool and authentication method in a future version of Galaxy (https://github.com/galaxyproject/galaxy/issues/14578).

**Figure 1:**
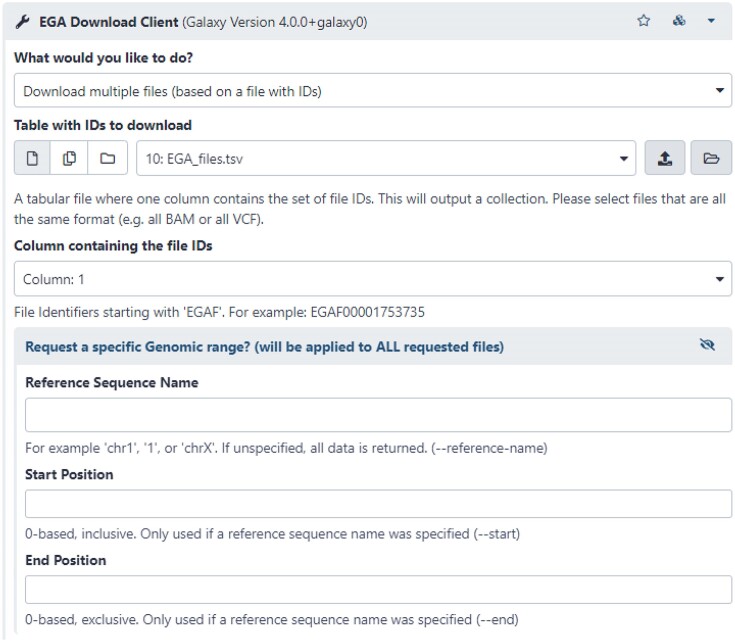
The Galaxy interface of the added feature to the PyEga3 tool to download multiple files. It takes a tabular data with EGAF IDs. In addition, a region can be provided to download a small region in BAMs or VCFs.

The tool implemented in Galaxy has the same functionalities as the command-line version—namely, list a user’s authorized datasets, list files in a dataset, and fetch a file or all files in a dataset. In addition, we added the option to download a specified list of files from the EGA. With this option, it is still possible to download a specific genomic range (see Fig. 1), which is useful for large binary alignment map (BAM) and variant calling format (VCF) files.

#### Gene.iobio

We also implemented the gene.iobio tool within the Galaxy framework. Gene.iobio is a tool for precision genomics. The tool is able to create dynamic results, which include creating a list of genes for the disease of interest, creating an automatic report of pathogenic variants within the list of genes, allowing the custom filtering of pathogenic variants, reporting phenotypes and publications related to the gene of interest, and reviewing the variants. These are major improvements compared to the existing trio analysis tool within Galaxy, GEMINI [17], which was only able to produce static plots or large lists of filtered variants.

#### Workflow and tutorial

In this study, we illustrate an end-to-end workflow for trio analysis for FAIR data. This workflow retrieves and analyzes files from large datasets in the EGA and can easily be adapted to any other EGA dataset. We illustrate this by analyzing data from the EGAD00001008392 [18] dataset. This dataset contains 6 trio families with different inheritance patterns of digitally spiked-in variants, where each family is subject to a different disease. Next, we demonstrate the utility of gene.iobio by analyzing the family trios and comparing the existing trio analysis tool in Galaxy, GEMINI, to the gene.iobio tool. Finally, a comprehensive tutorial is made available from the Galaxy Training materials [19] under the topic “Variant Analysis,” titled “Trio Analysis Using Synthetic Datasets from RD-Connect GPAP)” [20] to teach users how to access data from the EGA and to re-create and run the workflow from scratch. In addition, the workflow is available at WorkflowHub [21].

#### Use case: breast cancer

Here we report on the output produced by gene.iobio to demonstrate its added value to the Galaxy platform. To produce these results, we used case 5 from the EGA dataset. This case describes a family trio where the mother and daughter are affected by breast cancer. The case describes an autosomal dominant inheritance pattern, which causes a missense single-nucleotide polymorphism (SNP) at chromosome 17 position 41,215,920, changing a guanine into a thymine [18].

The BAMs and VCFs of the family trio are first downloaded using the PyEGA3 tool in Galaxy. The tool was able to securely download the trios’ VCFs and slices of the large BAMs by selecting chromosome 17. After downloading the data from the EGA, the workflow preprocesses the data and produces multiple outputs using gene.iobio.

First, a disease/phenotype of interest can be provided to produce a list of genes of interest. To generate this list of genes, the gene.iobio makes use of the Phenolyzer software [22]. In this case, the disease is breast cancer. The automatic selection of important genes related to the disease speeds up the process of finding causative variants. Alternatively, genes can be added manually.

**Figure 2:**
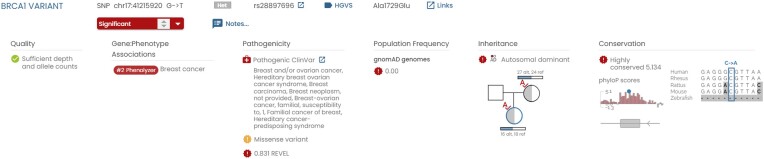
Overview of gene.iobio results for the spiked-in variant. The figure shows statistics on quality of the variant, phenotype associations, pathogenicity, population frequency, inheritance, and conservation.

Next, gene.iobio searches, by default, for causative variants in the top 20 of provided list of genes by filtering all the variants in the VCFs using preselected, but customizable, parameters. Fig. 2 shows that a spiked-in causative variant was found with sufficient depth and allele counts. In addition, gene.iobio shows the quality of the variant, a pathogenicity score, the population frequency, a visualization of the inheritance patterns, and statistics on the conservation of the variant. This information helps the user to determine the legitimacy of the variant.

Overall, gene.iobio provides an interactive and visual overview of causative variant identification. This is a significant improvement compared to the previous causative variant identification tool GEMINI, especially with regards to identifying the quality of the causative variant, as illustrated by Fig. 2.

#### Trio analysis comparison

In addition, we further validated the gene.iobio tool by identifying the causative variants in all the families available. A comparison of the results reported by gene.iobio and GEMINI is shown in Table 1. It shows the number of variants reported by GEMINI and gene.iobio using the default parameters. The table shows that GEMINI does not report any variants for some cases. When GEMINI does report variants, it reports the correct variants. However, it also reports many false positives, since each family has only 1 or 2 spiked-in causative variants. In contrast, gene.iobio does report causative variants for each family and only the correct ones. This shows that gene.iobio is not only interpretable but also accurate.

**Table 1:** Overview of existing Galaxy trio analysis tools and the number of variants they report

Family	GEMINI	Gene.iobio
Case 1	0	1
Case 2	77	1
Case 3	0	2
Case 4	26	1
Case 5	142	1
Case 6	0	1

## Limitations and Future Work

### PyEGA passports

In the current implementation of PyEGA3 in Galaxy, we miss the support of authentication with Passports, a Global Alliance for Genomics and Health (GA4GH) standard. The GA4GH has developed a set of standards to facilitate data sharing within a federated context. To access federated resources and controlled access data, the identity of the user accessing the data must be determined, along with any data access permissions the user has for particular datasets. Two GA4GH standards facilitate this, the AAI standard and the Passport standard. The AAI specification profiles OpenID Connect (OIDC) protocol provides a mechanism for interoperability of identities between different institutions, supporting federated data access while ensuring the security of the data by defining the way identities and access permissions are exchanged between resources. The Passport standard defines how the permissions are represented, in the form of visas. There are 5 types of visa: ControlledAccessGrants, which lists the access permissions for the user to controlled access datasets; LinkedIdentities, which allows a user to link different identities to facilitate single sign on; and AffiliationAndRole, AcceptedTermsAndPolicies, and ResearcherStatus. Passports support tiered access—open, registered, and controlled. Typically, the data available to the user will increase and the user moves from open to controlled access. Any user can access resources on the open access tier, while ResearcherStatus indicates the user can access resources at the registered access tier, and ControlledAccessGrants indicates which controlled access resources the user can access. The Life Science AAI supports GA4GH AAI and Passport standards. A user can link their Life Science identity with 1 or more institutional or social media identities and use these identities to access resources, such as Galaxy instances or datasets from the EGA. For example, a user can use their linked institutional identity via the Life Science AAI to access data from EGA via the EGA Permissions API and Data API. In the future, we aim to implement the Passport protocol into Galaxy to access data compliant with the GA4GH standards [23].

### Data management

In addition to secure data retrieval, Galaxy is working on improving secure data management. Currently, data are stored unencrypted on the user’s Galaxy account. But, with PyEGA implemented in Galaxy, confidential data from the EGA could be left unprotected when uploaded to a public Galaxy server. This would violate the EGA Data Access Agreement (DAA), which requires the user’s institution to preserve the confidentiality of the data.

Our approach for processing confidential human genetic data would currently only be in compliance with EGA guidelines and the General Data Protection Regulation [24] when data are stored on a private Galaxy managed by the user’s institution. However, data privacy at rest does exist within the community; currently, S3 buckets [25] can be leveraged by Galaxy, which offer the ability to encrypt data at rest. In the future, Galaxy’s Crypt4GH [26] integration project will provide a more deeply integrated alternative. This ongoing project aims to implement Crypt4GH [27], a standardized encryption tool for genetic data, to encrypt data at rest in Galaxy automatically. Currently, this project does not consider memory encryption as every individual tool that works with the data must implement support for trusted compute. Alternatively, directories could be encrypted with a user key, which would ensure the cached data are also encrypted.

### Data sharing

Once EGA data have been uploaded to a Galaxy instance, the data and analysis can easily be shared with other users. Currently, a user could share a history containing authenticated EGA data with another user who does not have DAC access, which is in violation of the DAA. Currently, Galaxy implements the ability to control privacy of individual datasets within a history, permitting the user to share the analysis results, without sharing the private source data. However, this remains a manual process. Therefore, we propose that in the future, the permission to share DAC access datasets to another Galaxy user is validated automatically. We suggest that this validation should be dataset specific (e.g., statistics, figures, and workflows derived from the history should be shareable as long as they ares in compliance with the DAA).

### Linking major repositories

In addition to the EGA, other major data repositories exist that have not been linked to Galaxy yet, such as The Cancer Genome Atlas Program (TCGA) [28]. The TCGA has its own data retrieval tool, the GDC Data Transfer Tool [29], for which separate credentials within a user’s Galaxy account have to be implemented. It would be more efficient for these repositories to support passports. This would greatly simplify the adoption of other major repositories in Galaxy in a GA4GH-compliant way, ultimately increasing the adoption of FAIR data principles.

An alternative to linking the repositories to Galaxy is to deploy a Galaxy instance where the data are stored. This would still require the Galaxy instance to download the data from the repository in the Galaxy instance, but the data will never leave the repository itself. However, this would require the data repository to have sufficient computing utilities in order to analyze the data with Galaxy. In the future, this might be a better alternative to linking the repositories itself.

## Conclusion

In this study, we implemented PyEGA3 in Galaxy to retrieve data from the EGA in a GA4GH-compliant manner. In addition, gene.iobio was implemented to improve variant analyses in Galaxy. These tools were validated by using B1MG data from the EGA and creating a findable analysis workflow into Galaxy. This work illustrates that gene.iobio is a major improvement compared to the current trio analysis tool in Galaxy as it creates interpretable and dynamic plots. In addition, we showed that Galaxy makes it feasible and manageable for any researcher to retrieve data from the EGA securely and analyze family trio data in a FAIR manner. Not only is this work applicable to trio analysis, but it is also transferable to other omics analysis, such as genome assembly, metabolomics, metagenomics, proteomics, and transcriptomics. In conclusion, this work illustrates that Galaxy is one step closer to becoming a generalized omics platform for FAIR data analysis.

## Methods

### Implementation

The installation and dependencies for gene.iobio are handled by Galaxy (RRID:SCR_006281). The version of gene.iobio reported here is v4.7.1a.

### Training materials

Our workflow simplifies the data collection from the EGA and the visualization and analysis of family trios. In addition, we created a tutorial for running the workflow on Galaxy. Also, the tutorial describes in detail how to gain access to datasets on the EGA to simplify the adoption of this workflow for other data on the EGA. The tutorial is available at the Galaxy training materials website [20].

### Preprocessing

First, a “chr” prefix is added to the first column of each chromosomal site in the VCFs, to match it with the built-in reference genome from Galaxy (hg19). Second, the VCFs are normalized using bcftools [30]. The normalization process includes left-aligning insertion or deletion and splitting multiallelic sites into biallelic records. Third, the VCFs in the EGA dataset are actually genomic variant calling format (GVCF) files. A GVCF has a record for (almost) all sites even when no variant, denoted by <NON_REF>, is recorded. In this study, this information is not informative and slows down analysis. Therefore, the records with a <NON_REF> site are filtered out. Fourth, the VCFs are merged into a single VCF based on their trio pairing using bcftools. This creates a VCF where each record also has a presence/absence column for each family pair. Finally, the variants in the merged VCF are filtered and annotated using the SnpEff tool [4] as required by GEMINI.

### Gene.iobio

Gene.iobio is run with the GRCh37 reference genome. The gene list is created using the phenotypes described in Additional file 1. The default search filters are used to detect the causative variant.

### GEMINI

For each case, GEMINI (RRID:SCR_014819) is prompted to remove low-impact severity variants and to search for causative variants that match the inheritance pattern in Additional file 1.

## Availability of Source Code and Requirements

### Galaxy resources

- Galaxy homepage: https://galaxyproject.org/
- Galaxy tutorials: https://training.galaxyproject.org
- How to install Galaxy: https://getgalaxy.org
- How to install tools: https://galaxyproject.org/admin/tools/add-tool-from-toolshed-tutorial/
- Full administrative resources: https://docs.galaxyproject.org/
- Galaxy Help Forum: https://help.galaxyproject.org/
- Connect with the Galaxy Community on Gitter Chat: https://gitter.im/galaxyproject/Lobby/

### ToolShed

- Project name: ToolShed repositories maintained by the Intergalactic Utilities Commission
- GitHub repository: https://github.com/galaxyproject/tools-iuc
- ToolShed repository: https://toolshed.g2.bx.psu.edu/
- License: MIT

### PyEGA3

- Project name: pyega3—uses the EGA REST API to download authorized datasets and files
- GitHub repository: https://github.com/galaxyproject/tools-iuc/tree/master/tools/pyega3
- ToolShed repository: https://toolshed.g2.bx.psu.edu/view/iuc/ega_download_client
- Training manual: https://training.galaxyproject.org/training-material/topics/variant-analysis/tutorials/trio-analysis/tutorial.html
- Operating system(s): Unix (Platform independent with Docker, Singularity)
- Other requirements: Galaxy version 22.05 or higher
- License: Apache-2.0
- RRID: SCR_024654

### Gene.iobio

- Project name: Gene.iobio—an interactive tool for variant and trio analysis
- GitHub repository: https://github.com/galaxyproject/tools-iuc/tree/master/tools/geneiobio
- ToolShed repository: https://toolshed.g2.bx.psu.edu/view/iuc/geneiobio
- Training manual: https://gxy.io/GTN:T00320
- Operating system(s): Unix (Platform independent with Docker, Singularity)
- Other requirements: Galaxy version 22.05 or higher
- License: MIT for academic use. Commercial use of gene.iobio is managed by Frameshift Labs who have been granted the exclusive commercial license by the University of Utah.

## Supplementary Material

giad099_GIGA-D-23-00177_Original_Submission

giad099_GIGA-D-23-00177_Revision_1

giad099_GIGA-D-23-00177_Revision_2

giad099_Response_to_Reviewer_Comments_Original_Submission

giad099_Response_to_Reviewer_Comments_Revision_1

giad099_Reviewer_1_Report_Original_Submission Federico Zambelli -- 7/24/2023 Reviewed

giad099_Reviewer_1_Report_Revision_1 Federico Zambelli -- 10/22/2023 Reviewed

giad099_Reviewer_2_Report_Original_Submission Timothy Griffin -- 9/3/2023 Reviewed

## Data Availability

The data used in this study were generated by a public human whole genome sequencing (WGS) experiment in the Illumina Platinum initiative [36], which was made available by the HapMap project [37]. All data from this project are available at the EGA website [12]. In this study, only the BAMs and VCFs with the chromosomes containing the spiked-in variants were included. These files are available under “RD-Connect GPAP Synthetic Data Spiked-in Variant Data” at Zenodo [38]. Other data further supporting this work are openly available in the *GigaScience* repository, GigaDB [39].

## Additional Files

Additional file 1. The report describing the family trios.

## Abbreviations

AAI: Authentication and Authorization; BAM: binary alignment map; B1MG: Beyond 1 Million Genomes; DAA: Data Access Agreement; DAC: Data Access Committee; EGA: European Genome-Phenome Archive; FAIR: Findable, Accessible, Interoperable, and Reusable; GA4GH: Global Alliance for Genomics and Health; GTN: Galaxy Training Network; GVCF: genomic variant calling format; OIDC: OpenID Connect; TCGA: The Cancer Genome Atlas Program; VCF: variant call format.
