# Supplementary material for: FAIR data retrieval for sensitive clinical research data in Galaxy
Source: Gigascience. 2024 Jan 27;13:giad099. doi: 10.1093/gigascience/giad099 (PMC10821763; doi:10.1093/gigascience/giad099)
Supplement: giad099_GIGA-D-23-00177_Original_Submission [file giad099_giga-d-23-00177_original_submission.pdf]

GigaScience

FAIR Data Retrieval for Sensitive Clinical Analysis in Galaxy

--Manuscript Draft--

**Manuscript Number:**	GIGA-D-23-00177
**Full Title:**	FAIR Data Retrieval for Sensitive Clinical Analysis in Galaxy
**Article Type:**	Technical Note
**Funding Information:**	Horizon 2020 (825775) — Dr Andrew Peter Stubbs<br>Erasmus+ (2020-1-NL01-KA203-064717) — Dr Andrew Peter Stubbs
**Abstract:**	Background, In clinical research, data has to be accessible and reproducible, but the generated data is becoming larger and analysis complex. Here we propose a platform for FAIR data access and creating reproducible findings. Standardised access to a major genomic repository, the European Genome-Phenome Archive (EGA), has been achieved with API services like PyEGA3. We aim to provide a FAIR data analysis service in Galaxy by retrieving genomic data from the EGA and provide a generalised “omics” platform for FAIR data analysis.<br>Results, To demonstrate this, we implemented an end-to-end Galaxy workflow to replicate the findings from an RD-Connect synthetic dataset Beyond the 1 Million Genomes (synB1MG) available from the EGA. We developed the PyEGA3 connector within Galaxy to easily download multiple datasets from the EGA. We added the gene.iobio tool, a diagnostic environment for precision genomics, to Galaxy and demonstrate that it provides a more dynamic and interpretable view for trio analysis results. We developed a Galaxy trio analysis workflow to determine the pathogenic variants from the synB1MG trios using the GEMINI and gene.iobio tool. The complete workflow is available at WorkflowHub and an associated tutorial was created in the Galaxy Training Network which helps researchers unfamiliar with Galaxy to run the workflow.<br>Conclusion, We showed the feasibility of reusing data from the EGA in Galaxy via PyEGA3 and validated the workflow by re-discovering spiked-in variants in synthetic data. Finally, we improved existing tools in Galaxy and created a workflow for trio analysis to demonstrate the value of FAIR genomics analysis in Galaxy.
**Corresponding Author:**	Jasper Ouwerkerk<br>Erasmus MC<br>Rotterdam, NETHERLANDS
**Corresponding Author Secondary Information:**	
**Corresponding Author's Institution:**	Erasmus MC
**Corresponding Author's Secondary Institution:**	
**First Author:**	Jasper Ouwerkerk
**First Author Secondary Information:**	
**Order of Authors:**	Jasper Ouwerkerk<br>Helena Rasche<br>John Dylan Spalding<br>Saskia Hiltemann<br>Andrew Peter Stubbs
**Order of Authors Secondary Information:**	
**Additional Information:**	
**Question**	**Response**

Powered by Editorial Manager® and ProduXion Manager® from Aries Systems Corporation

Are you submitting this manuscript to a special series or article collection?	No
**Experimental design and statistics**<br><br>Full details of the experimental design and statistical methods used should be given in the Methods section, as detailed in our Minimum Standards Reporting Checklist. Information essential to interpreting the data presented should be made available in the figure legends.<br><br>Have you included all the information requested in your manuscript?	Yes
**Resources**<br><br>A description of all resources used, including antibodies, cell lines, animals and software tools, with enough information to allow them to be uniquely identified, should be included in the Methods section. Authors are strongly encouraged to cite Research Resource Identifiers (RRIDs) for antibodies, model organisms and tools, where possible.<br><br>Have you included the information requested as detailed in our Minimum Standards Reporting Checklist?	Yes
**Availability of data and materials**<br><br>All datasets and code on which the conclusions of the paper rely must be either included in your submission or deposited in publicly available repositories (where available and ethically appropriate), referencing such data using a unique identifier in the references and in the "Availability of Data and Materials" section of your manuscript.<br><br>Have you have met the above requirement as detailed in our Minimum	Yes

Standards Reporting Checklist?	

Powered by Editorial Manager® and ProduXion Manager® from Aries Systems Corporation

LaTeX - Main Document (TeX file)
Click here to access/download;LaTeX - Main Document (TeX file);main.tex

```
This is pdfTeX, Version 3.141592653-2.6-1.40.24 (TeX Live 2022)
(preloaded format=pdflatex 2023.3.8)  28 JUN 2023 12:36
entering extended mode
 restricted \write18 enabled.
 %&-line parsing enabled.
**main.tex
(./main.tex
LaTeX2e <2022-11-01> patch level 1
L3 programming layer <2023-02-22> (./oup-contemporary.cls
Document Class: oup-contemporary 2017/06/28, v1.1
(c:/TeXLive/2022/texmf-dist/tex/latex/base/article.cls
Document Class: article 2022/07/02 v1.4n Standard LaTeX document class
(c:/TeXLive/2022/texmf-dist/tex/latex/base/size10.clo
File: size10.clo 2022/07/02 v1.4n Standard LaTeX file (size option)
)
\c@part=\count185
\c@section=\count186
\c@subsection=\count187
\c@subsubsection=\count188
\c@paragraph=\count189
\c@subparagraph=\count190
\c@figure=\count191
\c@table=\count192
\abovecaptionskip=\skip48
\belowcaptionskip=\skip49
\bibindent=\dimen140
) (c:/TeXLive/2022/texmf-dist/tex/latex/base/inputenc.sty
Package: inputenc 2021/02/14 v1.3d Input encoding file
\inpenc@prehook=\toks16
\inpenc@posthook=\toks17
) (c:/TeXLive/2022/texmf-dist/tex/latex/base/fontenc.sty
Package: fontenc 2021/04/29 v2.0v Standard LaTeX package
) (c:/TeXLive/2022/texmf-dist/tex/generic/iftex/ifpdf.sty
Package: ifpdf 2019/10/25 v3.4 ifpdf legacy package. Use iftex instead.
(c:/TeXLive/2022/texmf-dist/tex/generic/iftex/iftex.sty
Package: iftex 2022/02/03 v1.0f TeX engine tests
)) (c:/TeXLive/2022/texmf-dist/tex/latex/microtype/microtype.sty
Package: microtype 2023/03/13 v3.1a Micro-typographical refinements (RS)
(c:/TeXLive/2022/texmf-dist/tex/latex/graphics/keyval.sty
Package: keyval 2022/05/29 v1.15 key=value parser (DPC)
\KV@toks@=\toks18
) (c:/TeXLive/2022/texmf-dist/tex/latex/etoolbox/etoolbox.sty
Package: etoolbox 2020/10/05 v2.5k e-TeX tools for LaTeX (JAW)
\etb@tempcnta=\count193
)
\MT@toks=\toks19
\MT@tempbox=\box51
\MT@count=\count194
LaTeX Info: Redefining \noprotrusionifhmode on input line 1059.
LaTeX Info: Redefining \leftprotrusion on input line 1060.
\MT@prot@toks=\toks20
LaTeX Info: Redefining \rightprotrusion on input line 1078.
LaTeX Info: Redefining \textls on input line 1368.
\MT@outer@kern=\dimen141
```

```
LaTeX Info: Redefining \textmicrotypecontext on input line 1988.
\MT@listname@count=\count195
(c:/TeXLive/2022/texmf-dist/tex/latex/microtype/microtype-pdftex.def
File: microtype-pdftex.def 2023/03/13 v3.1a Definitions specific to
pdftex (RS)

LaTeX Info: Redefining \lsstyle on input line 902.
LaTeX Info: Redefining \lslig on input line 902.
\MT@outer@space=\skip50
)
Package microtype Info: Loading configuration file microtype.cfg.
(c:/TeXLive/2022/texmf-dist/tex/latex/microtype/microtype.cfg
File: microtype.cfg 2023/03/13 v3.1a microtype main configuration file
(RS)
)) (c:/TeXLive/2022/texmf-dist/tex/latex/euler/euler.sty
Package: euler 1995/03/05 v2.5
Package: `euler' v2.5 <1995/03/05> (FJ and FMi)
LaTeX Font Info:    Redeclaring symbol font `letters' on input line 35.
LaTeX Font Info:    Encoding `OML' has changed to `U' for symbol font
(Font)              `letters' in the math version `normal' on input line
35.
LaTeX Font Info:    Overwriting symbol font `letters' in version `normal'
(Font)                  OML/cmm/m/it --> U/eur/m/n on input line 35.
LaTeX Font Info:    Encoding `OML' has changed to `U' for symbol font
(Font)              `letters' in the math version `bold' on input line
35.
LaTeX Font Info:    Overwriting symbol font `letters' in version `bold'
(Font)                  OML/cmm/b/it --> U/eur/m/n on input line 35.
LaTeX Font Info:    Overwriting symbol font `letters' in version `bold'
(Font)                  U/eur/m/n --> U/eur/b/n on input line 36.
LaTeX Font Info:    Redeclaring math symbol \Gamma on input line 47.
LaTeX Font Info:    Redeclaring math symbol \Delta on input line 48.
LaTeX Font Info:    Redeclaring math symbol \Theta on input line 49.
LaTeX Font Info:    Redeclaring math symbol \Lambda on input line 50.
LaTeX Font Info:    Redeclaring math symbol \Xi on input line 51.
LaTeX Font Info:    Redeclaring math symbol \Pi on input line 52.
LaTeX Font Info:    Redeclaring math symbol \Sigma on input line 53.
LaTeX Font Info:    Redeclaring math symbol \Upsilon on input line 54.
LaTeX Font Info:    Redeclaring math symbol \Phi on input line 55.
LaTeX Font Info:    Redeclaring math symbol \Psi on input line 56.
LaTeX Font Info:    Redeclaring math symbol \Omega on input line 57.
\symEulerFraktur=\mathgroup4
LaTeX Font Info:    Overwriting symbol font `EulerFraktur' in version
`bold'
(Font)                  U/euf/m/n --> U/euf/b/n on input line 63.
LaTeX Info: Redefining \oldstylenums on input line 85.
\symEulerScript=\mathgroup5
LaTeX Font Info:    Overwriting symbol font `EulerScript' in version
`bold'
(Font)                  U/eus/m/n --> U/eus/b/n on input line 93.
LaTeX Font Info:    Redeclaring math symbol \aleph on input line 97.
LaTeX Font Info:    Redeclaring math symbol \Re on input line 98.
LaTeX Font Info:    Redeclaring math symbol \Im on input line 99.
LaTeX Font Info:    Redeclaring math delimiter \vert on input line 101.
```

```
LaTeX Font Info:    Redeclaring math delimiter \backslash on input line
103.
LaTeX Font Info:    Redeclaring math symbol \neg on input line 106.
LaTeX Font Info:    Redeclaring math symbol \wedge on input line 108.
LaTeX Font Info:    Redeclaring math symbol \vee on input line 110.
LaTeX Font Info:    Redeclaring math symbol \setminus on input line 112.
LaTeX Font Info:    Redeclaring math symbol \sim on input line 113.
LaTeX Font Info:    Redeclaring math symbol \mid on input line 114.
LaTeX Font Info:    Redeclaring math delimiter \arrowvert on input line
116.
LaTeX Font Info:    Redeclaring math symbol \mathsection on input line
117.
\symEulerExtension=\mathgroup6
LaTeX Font Info:    Redeclaring math symbol \coprod on input line 125.
LaTeX Font Info:    Redeclaring math symbol \prod on input line 125.
LaTeX Font Info:    Redeclaring math symbol \sum on input line 125.
LaTeX Font Info:    Redeclaring math symbol \intop on input line 130.
LaTeX Font Info:    Redeclaring math symbol \ointop on input line 131.
LaTeX Font Info:    Redeclaring math symbol \braceld on input line 132.
LaTeX Font Info:    Redeclaring math symbol \bracerd on input line 133.
LaTeX Font Info:    Redeclaring math symbol \bracelu on input line 134.
LaTeX Font Info:    Redeclaring math symbol \braceru on input line 135.
LaTeX Font Info:    Redeclaring math symbol \infty on input line 136.
LaTeX Font Info:    Redeclaring math symbol \nearrow on input line 153.
LaTeX Font Info:    Redeclaring math symbol \searrow on input line 154.
LaTeX Font Info:    Redeclaring math symbol \nwarrow on input line 155.
LaTeX Font Info:    Redeclaring math symbol \swarrow on input line 156.
LaTeX Font Info:    Redeclaring math symbol \Leftrightarrow on input line
157.
LaTeX Font Info:    Redeclaring math symbol \Leftarrow on input line 158.
LaTeX Font Info:    Redeclaring math symbol \Rightarrow on input line
159.
LaTeX Font Info:    Redeclaring math symbol \leftrightarrow on input line
160.
LaTeX Font Info:    Redeclaring math symbol \leftarrow on input line 161.
LaTeX Font Info:    Redeclaring math symbol \rightarrow on input line
163.
LaTeX Font Info:    Redeclaring math delimiter \uparrow on input line
166.
LaTeX Font Info:    Redeclaring math delimiter \downarrow on input line
168.
LaTeX Font Info:    Redeclaring math delimiter \updownarrow on input line
170.
LaTeX Font Info:    Redeclaring math delimiter \Uparrow on input line
172.
LaTeX Font Info:    Redeclaring math delimiter \Downarrow on input line
174.
LaTeX Font Info:    Redeclaring math delimiter \Updownarrow on input line
176.
LaTeX Font Info:    Redeclaring math symbol \leftharpoonup on input line
177.
LaTeX Font Info:    Redeclaring math symbol \leftharpoondown on input
line 178.
```

```
LaTeX Font Info:    Redeclaring math symbol \rightharpoonup on input line
179.
LaTeX Font Info:    Redeclaring math symbol \rightharpoondown on input
line 180
.
LaTeX Font Info:    Redeclaring math delimiter \lbrace on input line 182.
LaTeX Font Info:    Redeclaring math delimiter \rbrace on input line 184.
\symcmmigroup=\mathgroup7
LaTeX Font Info:    Overwriting symbol font `cmmigroup' in version `bold'
(Font)                  OML/cmm/m/it --> OML/cmm/b/it on input line 200.
LaTeX Font Info:    Redeclaring math accent \vec on input line 201.
LaTeX Font Info:    Redeclaring math symbol \triangleleft on input line
202.
LaTeX Font Info:    Redeclaring math symbol \triangleright on input line
203.
LaTeX Font Info:    Redeclaring math symbol \star on input line 204.
LaTeX Font Info:    Redeclaring math symbol \lhook on input line 205.
LaTeX Font Info:    Redeclaring math symbol \rhook on input line 206.
LaTeX Font Info:    Redeclaring math symbol \flat on input line 207.
LaTeX Font Info:    Redeclaring math symbol \natural on input line 208.
LaTeX Font Info:    Redeclaring math symbol \sharp on input line 209.
LaTeX Font Info:    Redeclaring math symbol \smile on input line 210.
LaTeX Font Info:    Redeclaring math symbol \frown on input line 211.
LaTeX Font Info:    Redeclaring math accent \grave on input line 245.
LaTeX Font Info:    Redeclaring math accent \acute on input line 246.
LaTeX Font Info:    Redeclaring math accent \tilde on input line 247.
LaTeX Font Info:    Redeclaring math accent \ddot on input line 248.
LaTeX Font Info:    Redeclaring math accent \check on input line 249.
LaTeX Font Info:    Redeclaring math accent \breve on input line 250.
LaTeX Font Info:    Redeclaring math accent \bar on input line 251.
LaTeX Font Info:    Redeclaring math accent \dot on input line 252.
LaTeX Font Info:    Redeclaring math accent \hat on input line 254.
) (c:/TeXLive/2022/texmf-dist/tex/latex/merriweather/merriweather.sty
Package: merriweather 2022/09/20 (Bob Tennent) Supports
Merriweather(Sans) font
s for all LaTeX engines.
(c:/TeXLive/2022/texmf-dist/tex/generic/iftex/ifxetex.sty
Package: ifxetex 2019/10/25 v0.7 ifxetex legacy package. Use iftex
instead.
) (c:/TeXLive/2022/texmf-dist/tex/generic/iftex/ifluatex.sty
Package: ifluatex 2019/10/25 v1.5 ifluatex legacy package. Use iftex
instead.
) (c:/TeXLive/2022/texmf-dist/tex/latex/base/textcomp.sty
Package: textcomp 2020/02/02 v2.0n Standard LaTeX package
) (c:/TeXLive/2022/texmf-dist/tex/latex/xkeyval/xkeyval.sty
Package: xkeyval 2022/06/16 v2.9 package option processing (HA)
(c:/TeXLive/2022/texmf-dist/tex/generic/xkeyval/xkeyval.tex
(c:/TeXLive/2022/te
xmf-dist/tex/generic/xkeyval/xkvutils.tex
\XKV@toks=\toks21
\XKV@tempa@toks=\toks22
)
\XKV@depth=\count196
File: xkeyval.tex 2014/12/03 v2.7a key=value parser (HA)
```

```
)) (c:/TeXLive/2022/texmf-dist/tex/latex/base/fontenc.sty
Package: fontenc 2021/04/29 v2.0v Standard LaTeX package
) (c:/TeXLive/2022/texmf-dist/tex/latex/fontaxes/fontaxes.sty
Package: fontaxes 2020/07/21 v1.0e Font selection axes
LaTeX Info: Redefining \upshape on input line 29.
LaTeX Info: Redefining \itshape on input line 31.
LaTeX Info: Redefining \slshape on input line 33.
LaTeX Info: Redefining \swshape on input line 35.
LaTeX Info: Redefining \scshape on input line 37.
LaTeX Info: Redefining \sscshape on input line 39.
LaTeX Info: Redefining \ulcshape on input line 41.
LaTeX Info: Redefining \textsw on input line 47.
LaTeX Info: Redefining \textssc on input line 48.
LaTeX Info: Redefining \textulc on input line 49.
)) (c:/TeXLive/2022/texmf-dist/tex/latex/mathastext/mathastext.sty
Package: mathastext 2022/11/04 v1.3y Use the text font in math mode (JFB)
\mst@exists@muskip=\muskip16
\mst@forall@muskip=\muskip17
\mst@prime@muskip=\muskip18
\mst@do@nonletters=\toks23
\mst@do@easynonletters=\toks24
\mst@do@az=\toks25
\mst@do@AZ=\toks26
\symmtoperatorfont=\mathgroup8
\symmtletterfont=\mathgroup9
** ! and ?
** punctuation: , . : ; and \colon
LaTeX Info: Redefining \relbar on input line 844.
LaTeX Info: Redefining \rightarrowfill on input line 847.
LaTeX Info: Redefining \leftarrowfill on input line 852.
** + and =
LaTeX Info: Redefining \Relbar on input line 943.
** adding = ; and + to \nfss@catcodes
** parentheses ( ) [ ] and slash /
** alldelims: < > \backslash \setminus | \vert \mid \{ and \}
LaTeX Font Info:    Redeclaring math delimiter \backslash on input line
989.
LaTeX Font Info:    Redeclaring math symbol \setminus on input line 1001.
LaTeX Info: Redefining \models on input line 1010.
** \# \mathdollar \% \&
** \imath and \jmath
LaTeX Font Info:    Overwriting math alphabet `\mathnormalbold' in
version `nor
mal'
(Font)                  T1/Merriwthr-OsF/b/it --> T1/Merriwthr-OsF/b/it
on inpu
t line 2370.
LaTeX Font Info:    Overwriting math alphabet `\mathnormalbold' in
version `bol
d'
(Font)                  T1/Merriwthr-OsF/b/it --> T1/Merriwthr-OsF/b/it
on inpu
t line 2370.
```

```
LaTeX Font Info:    Overwriting symbol font `mtletterfont' in version
`normal'
(Font)                  T1/Merriwthr-OsF/m/it --> T1/Merriwthr-OsF/m/it
on inpu
t line 2370.
LaTeX Font Info:    Overwriting symbol font `mtletterfont' in version
`bold'
(Font)                  T1/Merriwthr-OsF/m/it --> T1/Merriwthr-OsF/b/it
on inpu
t line 2370.
LaTeX Font Info:    Overwriting symbol font `mtoperatorfont' in version
`normal
'
(Font)                  T1/Merriwthr-OsF/m/n --> T1/Merriwthr-OsF/m/n on
input
line 2370.
LaTeX Font Info:    Overwriting symbol font `mtoperatorfont' in version
`bold'
(Font)                  T1/Merriwthr-OsF/m/n --> T1/Merriwthr-OsF/b/n on
input
line 2370.
LaTeX Font Info:    Overwriting math alphabet `\Mathbf' in version
`normal'
(Font)                  T1/Merriwthr-OsF/b/n --> T1/Merriwthr-OsF/b/n on
input
line 2370.
LaTeX Font Info:    Overwriting math alphabet `\Mathbf' in version `bold'
(Font)                  T1/Merriwthr-OsF/b/n --> T1/Merriwthr-OsF/b/n on
input
line 2370.
LaTeX Font Info:    Overwriting math alphabet `\Mathit' in version
`normal'
(Font)                  T1/Merriwthr-OsF/m/it --> T1/Merriwthr-OsF/m/it
on inpu
t line 2370.
LaTeX Font Info:    Overwriting math alphabet `\Mathit' in version `bold'
(Font)                  T1/Merriwthr-OsF/m/it --> T1/Merriwthr-OsF/b/it
on inpu
t line 2370.
LaTeX Font Info:    Overwriting math alphabet `\Mathsf' in version
`normal'
(Font)                  T1/MerriwthrSans-OsF/m/n --> T1/MerriwthrSans-
OsF/m/n o
n input line 2370.
LaTeX Font Info:    Overwriting math alphabet `\Mathsf' in version `bold'
(Font)                  T1/MerriwthrSans-OsF/m/n --> T1/MerriwthrSans-
OsF/b/n o
n input line 2370.
LaTeX Font Info:    Overwriting math alphabet `\Mathtt' in version
`normal'
(Font)                  T1/lmtt/m/n --> T1/lmtt/m/n on input line 2370.
LaTeX Font Info:    Overwriting math alphabet `\Mathtt' in version `bold'
(Font)                  T1/lmtt/m/n --> T1/lmtt/b/n on input line 2370.
** Latin letters in the `normal' (resp. `bold') math versions are now
```

```
** set up to use the fonts T1/Merriwthr-OsF/m(b)/it
** Other characters (digits, ...) and \log-like names will be
** typeset with the n shape.
** \hbar
** minus as endash
** \HUGE has been (re)-defined.
** mathastext has declared larger sizes for subscripts.
** To keep LaTeX defaults, use option `defaultmathsizes'.
) (c:/TeXLive/2022/texmf-dist/tex/latex/relsize/relsize.sty
Package: relsize 2013/03/29 ver 4.1
) (c:/TeXLive/2022/texmf-dist/tex/latex/ragged2e/ragged2e.sty
Package: ragged2e 2023/02/25 v3.4 ragged2e Package
\CenteringLeftskip=\skip51
\RaggedLeftLeftskip=\skip52
\RaggedRightLeftskip=\skip53
\CenteringRightskip=\skip54
\RaggedLeftRightskip=\skip55
\RaggedRightRightskip=\skip56
\CenteringParfillskip=\skip57
\RaggedLeftParfillskip=\skip58
\RaggedRightParfillskip=\skip59
\JustifyingParfillskip=\skip60
\CenteringParindent=\skip61
\RaggedLeftParindent=\skip62
\RaggedRightParindent=\skip63
\JustifyingParindent=\skip64
) (c:/TeXLive/2022/texmf-dist/tex/latex/xcolor/xcolor.sty
Package: xcolor 2022/06/12 v2.14 LaTeX color extensions (UK)
(c:/TeXLive/2022/texmf-dist/tex/latex/graphics-cfg/color.cfg
File: color.cfg 2016/01/02 v1.6 sample color configuration
)
Package xcolor Info: Driver file: pdftex.def on input line 227.
(c:/TeXLive/2022/texmf-dist/tex/latex/graphics-def/pdftex.def
File: pdftex.def 2022/09/22 v1.2b Graphics/color driver for pdftex
) (c:/TeXLive/2022/texmf-dist/tex/latex/graphics/mathcolor.ltx)
Package xcolor Info: Model `cmy' substituted by `cmy0' on input line
1353.
Package xcolor Info: Model `hsb' substituted by `rgb' on input line 1357.
Package xcolor Info: Model `RGB' extended on input line 1369.
Package xcolor Info: Model `HTML' substituted by `rgb' on input line
1371.
Package xcolor Info: Model `Hsb' substituted by `hsb' on input line 1372.
Package xcolor Info: Model `tHsb' substituted by `hsb' on input line
1373.
Package xcolor Info: Model `HSB' substituted by `hsb' on input line 1374.
Package xcolor Info: Model `Gray' substituted by `gray' on input line
1375.
Package xcolor Info: Model `wave' substituted by `hsb' on input line
1376.
) (c:/TeXLive/2022/texmf-dist/tex/latex/colortbl/colortbl.sty
Package: colortbl 2022/06/20 v1.0f Color table columns (DPC)
(c:/TeXLive/2022/texmf-dist/tex/latex/tools/array.sty
Package: array 2022/09/04 v2.5g Tabular extension package (FMi)
\col@sep=\dimen142
```

```
\ar@mcellbox=\box52
\extrarowheight=\dimen143
\NC@list=\toks27
\extratabsurround=\skip65
\backup@length=\skip66
\ar@cellbox=\box53
)
\everycr=\toks28
\minrowclearance=\skip67
\rownum=\count197
) (c:/TeXLive/2022/texmf-dist/tex/latex/graphics/graphicx.sty
Package: graphicx 2021/09/16 v1.2d Enhanced LaTeX Graphics (DPC,SPQR)
(c:/TeXLive/2022/texmf-dist/tex/latex/graphics/graphics.sty
Package: graphics 2022/03/10 v1.4e Standard LaTeX Graphics (DPC,SPQR)
(c:/TeXLive/2022/texmf-dist/tex/latex/graphics/trig.sty
Package: trig 2021/08/11 v1.11 sin cos tan (DPC)
) (c:/TeXLive/2022/texmf-dist/tex/latex/graphics-cfg/graphics.cfg
File: graphics.cfg 2016/06/04 v1.11 sample graphics configuration
)
Package graphics Info: Driver file: pdftex.def on input line 107.
)
\Gin@req@height=\dimen144
\Gin@req@width=\dimen145
) (c:/TeXLive/2022/texmf-dist/tex/latex/xpatch/xpatch.sty
(c:/TeXLive/2022/texm
f-dist/tex/latex/l3kernel/expl3.sty
Package: expl3 2023-02-22 L3 programming layer (loader)
(c:/TeXLive/2022/texmf-dist/tex/latex/l3backend/l3backend-pdftex.def
File: l3backend-pdftex.def 2023-01-16 L3 backend support: PDF output
(pdfTeX)
\l__color_backend_stack_int=\count198
\l__pdf_internal_box=\box54
))
Package: xpatch 2020/03/25 v0.3a Extending etoolbox patching commands
(c:/TeXLive/2022/texmf-dist/tex/latex/l3packages/xparse/xparse.sty
Package: xparse 2023-02-02 L3 Experimental document command parser
)) (c:/TeXLive/2022/texmf-dist/tex/latex/environ/environ.sty
Package: environ 2014/05/04 v0.3 A new way to define environments
(c:/TeXLive/2022/texmf-dist/tex/latex/trimspaces/trimspaces.sty
Package: trimspaces 2009/09/17 v1.1 Trim spaces around a token list
)
\@envbody=\toks29
) (c:/TeXLive/2022/texmf-dist/tex/latex/lastpage/lastpage.sty
Package: lastpage 2023/03/07 v2.0a lastpage: 2.09 or 2e? (HMM)
(c:/TeXLive/2022/texmf-dist/tex/latex/lastpage/lastpage2e.sty
Package: lastpage2e 2023/03/07 v2.0a Decide which 2e lastpage version to
use (H
MM)
(c:/TeXLive/2022/texmf-dist/tex/latex/lastpage/lastpagemodern.sty
Package: lastpagemodern 2023-03-07 v2.0a Refers to last page's name (HMM;
JPG)
)
)) (c:/TeXLive/2022/texmf-dist/tex/latex/graphics/rotating.sty
Package: rotating 2016/08/11 v2.16d rotated objects in LaTeX
```

```
(c:/TeXLive/2022/texmf-dist/tex/latex/base/ifthen.sty
Package: ifthen 2022/04/13 v1.1d Standard LaTeX ifthen package (DPC)
)
\c@r@tfl@t=\count199
\rotFPtop=\skip68
\rotFPbot=\skip69
\rot@float@box=\box55
\rot@mess@toks=\toks30
) (c:/TeXLive/2022/texmf-dist/tex/latex/graphics/lscape.sty
Package: lscape 2020/05/28 v3.02 Landscape Pages (DPC)
) (c:/TeXLive/2022/texmf-dist/tex/latex/tools/afterpage.sty
Package: afterpage 2014/10/28 v1.08 After-Page Package (DPC)
\AP@output=\toks31
\AP@partial=\box56
\AP@footins=\box57
) (c:/TeXLive/2022/texmf-dist/tex/latex/textpos/textpos.sty
Package: textpos 2022/07/23 v1.10.1
Package textpos Info: choosing support for LaTeX3 on input line 60.
\TP@textbox=\box58
\TP@holdbox=\box59
\TPHorizModule=\dimen146
\TPVertModule=\dimen147
\TP@margin=\dimen148
\TP@absmargin=\dimen149
Grid set 16 x 16 = 37.34424pt x 52.81541pt
\TPboxrulesize=\dimen150
\TP@ox=\dimen151
\TP@oy=\dimen152
\TP@tbargs=\toks32
TextBlockOrigin set to 0pt x 0pt
) (c:/TeXLive/2022/texmf-dist/tex/latex/url/url.sty
\Urlmuskip=\muskip19
Package: url 2013/09/16  ver 3.4  Verb mode for urls, etc.
) (c:/TeXLive/2022/texmf-dist/tex/latex/newfloat/newfloat.sty
Package: newfloat 2019/09/02 v1.1l Defining new floating environments
(AR)
Package newfloat Info: `rotating' package detected.
) (c:/TeXLive/2022/texmf-dist/tex/latex/mdframed/mdframed.sty
Package: mdframed 2013/07/01 1.9b: mdframed
(c:/TeXLive/2022/texmf-dist/tex/latex/kvoptions/kvoptions.sty
Package: kvoptions 2022-06-15 v3.15 Key value format for package options
(HO)
(c:/TeXLive/2022/texmf-dist/tex/generic/ltxcmds/ltxcmds.sty
Package: ltxcmds 2020-05-10 v1.25 LaTeX kernel commands for general use
(HO)
) (c:/TeXLive/2022/texmf-dist/tex/latex/kvsetkeys/kvsetkeys.sty
Package: kvsetkeys 2022-10-05 v1.19 Key value parser (HO)
)) (c:/TeXLive/2022/texmf-dist/tex/latex/zref/zref-abspage.sty
Package: zref-abspage 2022-04-07 v2.34 Module abspage for zref (HO)
(c:/TeXLive/2022/texmf-dist/tex/latex/zref/zref-base.sty
Package: zref-base 2022-04-07 v2.34 Module base for zref (HO)
(c:/TeXLive/2022/texmf-dist/tex/generic/infwarerr/infwarerr.sty
Package: infwarerr 2019/12/03 v1.5 Providing info/warning/error messages
(HO)
```

```
) (c:/TeXLive/2022/texmf-dist/tex/generic/kvdefinekeys/kvdefinekeys.sty
Package: kvdefinekeys 2019-12-19 v1.6 Define keys (HO)
) (c:/TeXLive/2022/texmf-dist/tex/generic/pdftexcmds/pdftexcmds.sty
Package: pdftexcmds 2020-06-27 v0.33 Utility functions of pdfTeX for
LuaTeX (HO
)
Package pdftexcmds Info: \pdf@primitive is available.
Package pdftexcmds Info: \pdf@ifprimitive is available.
Package pdftexcmds Info: \pdfdraftmode found.
) (c:/TeXLive/2022/texmf-dist/tex/generic/etexcmds/etexcmds.sty
Package: etexcmds 2019/12/15 v1.7 Avoid name clashes with e-TeX commands
(HO)
) (c:/TeXLive/2022/texmf-dist/tex/latex/auxhook/auxhook.sty
Package: auxhook 2019-12-17 v1.6 Hooks for auxiliary files (HO)
)
Package zref Info: New property list: main on input line 767.
Package zref Info: New property: default on input line 768.
Package zref Info: New property: page on input line 769.
) (c:/TeXLive/2022/texmf-dist/tex/latex/base/atbegshi-ltx.sty
Package: atbegshi-ltx 2021/01/10 v1.0c Emulation of the original atbegshi
package with kernel methods
)
\c@abspage=\count266
Package zref Info: New property: abspage on input line 65.
) (c:/TeXLive/2022/texmf-dist/tex/latex/needspace/needspace.sty
Package: needspace 2010/09/12 v1.3d reserve vertical space
)
\mdf@templength=\skip70
\c@mdf@globalstyle@cnt=\count267
\mdf@skipabove@length=\skip71
\mdf@skipbelow@length=\skip72
\mdf@leftmargin@length=\skip73
\mdf@rightmargin@length=\skip74
\mdf@innerleftmargin@length=\skip75
\mdf@innerrightmargin@length=\skip76
\mdf@innertopmargin@length=\skip77
\mdf@innerbottommargin@length=\skip78
\mdf@splittopskip@length=\skip79
\mdf@splitbottomskip@length=\skip80
\mdf@outermargin@length=\skip81
\mdf@innermargin@length=\skip82
\mdf@linewidth@length=\skip83
\mdf@innerlinewidth@length=\skip84
\mdf@middlelinewidth@length=\skip85
\mdf@outerlinewidth@length=\skip86
\mdf@roundcorner@length=\skip87
\mdf@footenotedistance@length=\skip88
\mdf@userdefinedwidth@length=\skip89
\mdf@needspace@length=\skip90
\mdf@frametitleaboveskip@length=\skip91
\mdf@frametitlebelowskip@length=\skip92
\mdf@frametitlerulewidth@length=\skip93
\mdf@frametitleleftmargin@length=\skip94
\mdf@frametitlerightmargin@length=\skip95
```

```
\mdf@shadowsize@length=\skip96
\mdf@extratopheight@length=\skip97
\mdf@subtitleabovelinewidth@length=\skip98
\mdf@subtitlebelowlinewidth@length=\skip99
\mdf@subtitleaboveskip@length=\skip100
\mdf@subtitlebelowskip@length=\skip101
\mdf@subtitleinneraboveskip@length=\skip102
\mdf@subtitleinnerbelowskip@length=\skip103
\mdf@subsubtitleabovelinewidth@length=\skip104
\mdf@subsubtitlebelowlinewidth@length=\skip105
\mdf@subsubtitleaboveskip@length=\skip106
\mdf@subsubtitlebelowskip@length=\skip107
\mdf@subsubtitleinneraboveskip@length=\skip108
\mdf@subsubtitleinnerbelowskip@length=\skip109
(c:/TeXLive/2022/texmf-dist/tex/latex/mdframed/md-frame-0.mdf
File: md-frame-0.mdf 2013/07/01\ 1.9b: md-frame-0
)
\mdf@frametitlebox=\box60
\mdf@footnotebox=\box61
\mdf@splitbox@one=\box62
\mdf@splitbox@two=\box63
\mdf@splitbox@save=\box64
\mdfsplitboxwidth=\skip110
\mdfsplitboxtotalwidth=\skip111
\mdfsplitboxheight=\skip112
\mdfsplitboxdepth=\skip113
\mdfsplitboxtotalheight=\skip114
\mdfframetitleboxwidth=\skip115
\mdfframetitleboxtotalwidth=\skip116
\mdfframetitleboxheight=\skip117
\mdfframetitleboxdepth=\skip118
\mdfframetitleboxtotalheight=\skip119
\mdffootnoteboxwidth=\skip120
\mdffootnoteboxtotalwidth=\skip121
\mdffootnoteboxheight=\skip122
\mdffootnoteboxdepth=\skip123
\mdffootnoteboxtotalheight=\skip124
\mdftotallinewidth=\skip125
\mdfboundingboxwidth=\skip126
\mdfboundingboxtotalwidth=\skip127
\mdfboundingboxheight=\skip128
\mdfboundingboxdepth=\skip129
\mdfboundingboxtotalheight=\skip130
\mdf@freevspace@length=\skip131
\mdf@horizontalwidthofbox@length=\skip132
\mdf@verticalmarginwhole@length=\skip133
\mdf@horizontalspaceofbox=\skip134
\mdfsubtitleheight=\skip135
\mdfsubsubtitleheight=\skip136
\c@mdfcountframes=\count268

****** mdframed patching \endmdf@trivlist

****** -- success******
```

```
\mdf@envdepth=\count269
\c@mdf@env@i=\count270
\c@mdf@env@ii=\count271
\c@mdf@zref@counter=\count272
Package zref Info: New property: mdf@pagevalue on input line 895.
) (c:/TeXLive/2022/texmf-dist/tex/latex/titlesec/titlesec.sty
Package: titlesec 2021/07/05 v2.14 Sectioning titles
\ttl@box=\box65
\beforetitleunit=\skip137
\aftertitleunit=\skip138
\ttl@plus=\dimen153
\ttl@minus=\dimen154
\ttl@toksa=\toks33
\titlewidth=\dimen155
\titlewidthlast=\dimen156
\titlewidthfirst=\dimen157
) (c:/TeXLive/2022/texmf-dist/tex/latex/koma-script/scrextend.sty
Package: scrextend 2022/10/12 v3.38 KOMA-Script package (extend other
classes w
ith features of KOMA-Script classes)
(c:/TeXLive/2022/texmf-dist/tex/latex/koma-script/scrkbase.sty
Package: scrkbase 2022/10/12 v3.38 KOMA-Script package (KOMA-Script-
dependent b
asics and keyval usage)
(c:/TeXLive/2022/texmf-dist/tex/latex/koma-script/scrbase.sty
Package: scrbase 2022/10/12 v3.38 KOMA-Script package (KOMA-Script-
independent
basics and keyval usage)
(c:/TeXLive/2022/texmf-dist/tex/latex/koma-script/scrlfile.sty
Package: scrlfile 2022/10/12 v3.38 KOMA-Script package (file load hooks)
(c:/TeXLive/2022/texmf-dist/tex/latex/koma-script/scrlfile-hook.sty
Package: scrlfile-hook 2022/10/12 v3.38 KOMA-Script package (using LaTeX
hooks)

(c:/TeXLive/2022/texmf-dist/tex/latex/koma-script/scrlogo.sty
Package: scrlogo 2022/10/12 v3.38 KOMA-Script package (logo)
)))
Applying: [2021/05/01] Usage of raw or classic option list on input line
252.
Already applied: [0000/00/00] Usage of raw or classic option list on
input line
 368.
))
Package scrextend Info: unexpected definition of `\@makefnmark'.
(scrextend)             Trying to patch it on input line 1709.
Package scrextend Info: patch seems to be successfull on input line 1709.
)

LaTeX Font Warning: Font shape `T1/cmr/m/n' in size <7.5> not available
(Font)              size <7> substituted on input line 65.

(c:/TeXLive/2022/texmf-dist/tex/latex/tools/calc.sty
Package: calc 2017/05/25 v4.3 Infix arithmetic (KKT,FJ)
```

```
\calc@Acount=\count273
\calc@Bcount=\count274
\calc@Adimen=\dimen158
\calc@Bdimen=\dimen159
\calc@Askip=\skip139
\calc@Bskip=\skip140
LaTeX Info: Redefining \setlength on input line 80.
LaTeX Info: Redefining \addtolength on input line 81.
\calc@Ccount=\count275
\calc@Cskip=\skip141
) (c:/TeXLive/2022/texmf-dist/tex/latex/geometry/geometry.sty
Package: geometry 2020/01/02 v5.9 Page Geometry
(c:/TeXLive/2022/texmf-dist/tex/generic/iftex/ifvtex.sty
Package: ifvtex 2019/10/25 v1.7 ifvtex legacy package. Use iftex instead.
)
\Gm@cnth=\count276
\Gm@cntv=\count277
\c@Gm@tempcnt=\count278
\Gm@bindingoffset=\dimen160
\Gm@wd@mp=\dimen161
\Gm@odd@mp=\dimen162
\Gm@even@mp=\dimen163
\Gm@layoutwidth=\dimen164
\Gm@layoutheight=\dimen165
\Gm@layouthoffset=\dimen166
\Gm@layoutvoffset=\dimen167
\Gm@dimlist=\toks34
) (c:/TeXLive/2022/texmf-dist/tex/latex/hyperref/hyperref.sty
Package: hyperref 2023-02-07 v7.00v Hypertext links for LaTeX
(c:/TeXLive/2022/texmf-dist/tex/generic/pdfescape/pdfescape.sty
Package: pdfescape 2019/12/09 v1.15 Implements pdfTeX's escape features
(HO)
) (c:/TeXLive/2022/texmf-dist/tex/latex/hycolor/hycolor.sty
Package: hycolor 2020-01-27 v1.10 Color options for hyperref/bookmark
(HO)
) (c:/TeXLive/2022/texmf-dist/tex/latex/letltxmacro/letltxmacro.sty
Package: letltxmacro 2019/12/03 v1.6 Let assignment for LaTeX macros (HO)
) (c:/TeXLive/2022/texmf-dist/tex/latex/hyperref/nameref.sty
Package: nameref 2022-05-17 v2.50 Cross-referencing by name of section
(c:/TeXLive/2022/texmf-dist/tex/latex/refcount/refcount.sty
Package: refcount 2019/12/15 v3.6 Data extraction from label references
(HO)
) (c:/TeXLive/2022/texmf-
dist/tex/generic/gettitlestring/gettitlestring.sty
Package: gettitlestring 2019/12/15 v1.6 Cleanup title references (HO)
)
\c@section@level=\count279
)
\@linkdim=\dimen168
\Hy@linkcounter=\count280
\Hy@pagecounter=\count281
(c:/TeXLive/2022/texmf-dist/tex/latex/hyperref/pd1enc.def
File: pd1enc.def 2023-02-07 v7.00v Hyperref: PDFDocEncoding definition
(HO)
```

```
Now handling font encoding PD1 ...
... no UTF-8 mapping file for font encoding PD1
) (c:/TeXLive/2022/texmf-dist/tex/generic/intcalc/intcalc.sty
Package: intcalc 2019/12/15 v1.3 Expandable calculations with integers
(HO)
)
\Hy@SavedSpaceFactor=\count282
(c:/TeXLive/2022/texmf-dist/tex/latex/hyperref/puenc.def
File: puenc.def 2023-02-07 v7.00v Hyperref: PDF Unicode definition (HO)
Now handling font encoding PU ...
... no UTF-8 mapping file for font encoding PU
)
Package hyperref Info: Option `colorlinks' set `true' on input line 4060.
Package hyperref Info: Hyper figures OFF on input line 4177.
Package hyperref Info: Link nesting OFF on input line 4182.
Package hyperref Info: Hyper index ON on input line 4185.
Package hyperref Info: Plain pages OFF on input line 4192.
Package hyperref Info: Backreferencing OFF on input line 4197.
Package hyperref Info: Implicit mode ON; LaTeX internals redefined.
Package hyperref Info: Bookmarks ON on input line 4425.
\c@Hy@tempcnt=\count283
LaTeX Info: Redefining \url on input line 4763.
\XeTeXLinkMargin=\dimen169
(c:/TeXLive/2022/texmf-dist/tex/generic/bitset/bitset.sty
Package: bitset 2019/12/09 v1.3 Handle bit-vector datatype (HO)
(c:/TeXLive/2022/texmf-dist/tex/generic/bigintcalc/bigintcalc.sty
Package: bigintcalc 2019/12/15 v1.5 Expandable calculations on big
integers (HO
)
))
\Fld@menulength=\count284
\Field@Width=\dimen170
\Fld@charsize=\dimen171
Package hyperref Info: Hyper figures OFF on input line 6042.
Package hyperref Info: Link nesting OFF on input line 6047.
Package hyperref Info: Hyper index ON on input line 6050.
Package hyperref Info: backreferencing OFF on input line 6057.
Package hyperref Info: Link coloring ON on input line 6060.
Package hyperref Info: Link coloring with OCG OFF on input line 6067.
Package hyperref Info: PDF/A mode OFF on input line 6072.
\Hy@abspage=\count285
\c@Item=\count286
\c@Hfootnote=\count287
)
Package hyperref Info: Driver (autodetected): hpdftex.
(c:/TeXLive/2022/texmf-dist/tex/latex/hyperref/hpdftex.def
File: hpdftex.def 2023-02-07 v7.00v Hyperref driver for pdfTeX
(c:/TeXLive/2022/texmf-dist/tex/latex/base/atveryend-ltx.sty
Package: atveryend-ltx 2020/08/19 v1.0a Emulation of the original
atveryend pac
kage
with kernel methods
)
\HyAnn@Count=\count288
```

```
\Fld@listcount=\count289
\c@bookmark@seq@number=\count290
(c:/TeXLive/2022/texmf-dist/tex/latex/rerunfilecheck/rerunfilecheck.sty
Package: rerunfilecheck 2022-07-10 v1.10 Rerun checks for auxiliary files
(HO)
(c:/TeXLive/2022/texmf-dist/tex/generic/uniquecounter/uniquecounter.sty
Package: uniquecounter 2019/12/15 v1.4 Provide unlimited unique counter
(HO)
)
Package uniquecounter Info: New unique counter `rerunfilecheck' on input
line 2
85.
)
\Hy@SectionHShift=\skip142
) (c:/TeXLive/2022/texmf-dist/tex/latex/preprint/authblk.sty
Package: authblk 2001/02/27 1.3 (PWD)
\affilsep=\skip143
\@affilsep=\skip144
\c@Maxaffil=\count291
\c@authors=\count292
\c@affil=\count293
) (c:/TeXLive/2022/texmf-dist/tex/latex/footmisc/footmisc.sty
Package: footmisc 2022/03/08 v6.0d a miscellany of footnote facilities
\FN@temptoken=\toks35
\footnotemargin=\dimen172
\@outputbox@depth=\dimen173
Package footmisc Info: Declaring symbol style bringhurst on input line
695.
Package footmisc Info: Declaring symbol style chicago on input line 703.
Package footmisc Info: Declaring symbol style wiley on input line 712.
Package footmisc Info: Declaring symbol style lamport-robust on input
line 723.

Package footmisc Info: Declaring symbol style lamport* on input line 743.
Package footmisc Info: Declaring symbol style lamport*-robust on input
line 764
.
) (c:/TeXLive/2022/texmf-dist/tex/latex/fancyhdr/fancyhdr.sty
Package: fancyhdr 2022/11/09 v4.1 Extensive control of page headers and
footers

\f@nch@headwidth=\skip145
\f@nch@O@elh=\skip146
\f@nch@O@erh=\skip147
\f@nch@O@olh=\skip148
\f@nch@O@orh=\skip149
\f@nch@O@elf=\skip150
\f@nch@O@erf=\skip151
\f@nch@O@olf=\skip152
\f@nch@O@orf=\skip153
) (c:/TeXLive/2022/texmf-dist/tex/generic/alphalph/alphalph.sty
Package: alphalph 2019/12/09 v2.6 Convert numbers to letters (HO)
)
\c@authorfn=\count294
```

```
(c:/TeXLive/2022/texmf-dist/tex/latex/abstract/abstract.sty
Package: abstract 2009/06/08 v1.2a configurable abstracts
\abstitleskip=\skip154
\absleftindent=\skip155
\absrightindent=\skip156
\absparindent=\skip157
\absparsep=\skip158
)
Package newfloat Info: New float `keypoints' with options
`placement=t!,name=kp
t' on input line 286.
\c@keypoints=\count295
\newfloat@ftype=\count296
Package newfloat Info: float type `keypoints'=8 on input line 286.
(c:/TeXLive/2022/texmf-dist/tex/latex/enumitem/enumitem.sty
Package: enumitem 2019/06/20 v3.9 Customized lists
\labelindent=\skip159
\enit@outerparindent=\dimen174
\enit@toks=\toks36
\enit@inbox=\box66
\enit@count@id=\count297
\enitdp@description=\count298
) (c:/TeXLive/2022/texmf-dist/tex/latex/quoting/quoting.sty
Package: quoting 2014/01/28 v0.1c Consolidated environment for displayed
text
\quo@toppartop=\skip160
) (c:/TeXLive/2022/texmf-dist/tex/latex/sttools/stfloats.sty
Package: stfloats 2017/03/27 v3.3 Improve float mechanism and
baselineskip sett
ings
\@dblbotnum=\count299
\c@dblbotnumber=\count300
) (c:/TeXLive/2022/texmf-dist/tex/latex/booktabs/booktabs.sty
Package: booktabs 2020/01/12 v1.61803398 Publication quality tables
\heavyrulewidth=\dimen175
\lightrulewidth=\dimen176
\cmidrulewidth=\dimen177
\belowrulesep=\dimen178
\belowbottomsep=\dimen179
\aboverulesep=\dimen180
\abovetopsep=\dimen181
\cmidrulesep=\dimen182
\cmidrulekern=\dimen183
\defaultaddspace=\dimen184
\@cmidla=\count301
\@cmidlb=\count302
\@aboverulesep=\dimen185
\@belowrulesep=\dimen186
\@thisruleclass=\count303
\@lastruleclass=\count304
\@thisrulewidth=\dimen187
) (c:/TeXLive/2022/texmf-dist/tex/latex/tools/tabularx.sty
Package: tabularx 2020/01/15 v2.11c `tabularx' package (DPC)
\TX@col@width=\dimen188
```

```
\TX@old@table=\dimen189
\TX@old@col=\dimen190
\TX@target=\dimen191
\TX@delta=\dimen192
\TX@cols=\count305
\TX@ftn=\toks37
)
\enitdp@tablenotes=\count306
(c:/TeXLive/2022/texmf-dist/tex/latex/caption/caption.sty
Package: caption 2022/03/01 v3.6b Customizing captions (AR)
(c:/TeXLive/2022/texmf-dist/tex/latex/caption/caption3.sty
Package: caption3 2022/03/17 v2.3b caption3 kernel (AR)
\caption@tempdima=\dimen193
\captionmargin=\dimen194
\caption@leftmargin=\dimen195
\caption@rightmargin=\dimen196
\caption@width=\dimen197
\caption@indent=\dimen198
\caption@parindent=\dimen199
\caption@hangindent=\dimen256
Package caption Info: Standard document class detected.
)
\c@caption@flags=\count307
\c@continuedfloat=\count308
Package caption Info: hyperref package is loaded.
Package caption Info: rotating package is loaded.
) (c:/TeXLive/2022/texmf-dist/tex/latex/natbib/natbib.sty
Package: natbib 2010/09/13 8.31b (PWD, AO)
\bibhang=\skip161
\bibsep=\skip162
LaTeX Info: Redefining \cite on input line 694.
\c@NAT@ctr=\count309
)) (c:/TeXLive/2022/texmf-dist/tex/latex/siunitx/siunitx.sty
Package: siunitx 2023-03-04 v3.2.2 A comprehensive (SI) units package
\l__siunitx_angle_tmp_dim=\dimen257
\l__siunitx_angle_marker_box=\box67
\l__siunitx_angle_unit_box=\box68
\l__siunitx_compound_count_int=\count310
(c:/TeXLive/2022/texmf-dist/tex/latex/translations/translations.sty
Package: translations 2022/02/05 v1.12 internationalization of LaTeX2e
packages
 (CN)
)
\l__siunitx_number_exponent_fixed_int=\count311
\l__siunitx_number_min_decimal_int=\count312
\l__siunitx_number_min_integer_int=\count313
\l__siunitx_number_round_precision_int=\count314
\l__siunitx_number_lower_threshold_int=\count315
\l__siunitx_number_upper_threshold_int=\count316
\l__siunitx_number_group_first_int=\count317
\l__siunitx_number_group_size_int=\count318
\l__siunitx_number_group_minimum_int=\count319
(c:/TeXLive/2022/texmf-dist/tex/latex/amsmath/amstext.sty
Package: amstext 2021/08/26 v2.01 AMS text
```

```
(c:/TeXLive/2022/texmf-dist/tex/latex/amsmath/amsgen.sty
File: amsgen.sty 1999/11/30 v2.0 generic functions
\@emptytoks=\toks38
\ex@=\dimen258
))
\l__siunitx_table_tmp_box=\box69
\l__siunitx_table_tmp_dim=\dimen259
\l__siunitx_table_column_width_dim=\dimen260
\l__siunitx_table_integer_box=\box70
\l__siunitx_table_decimal_box=\box71
\l__siunitx_table_uncert_box=\box72
\l__siunitx_table_before_box=\box73
\l__siunitx_table_after_box=\box74
\l__siunitx_table_before_dim=\dimen261
\l__siunitx_table_carry_dim=\dimen262
\l__siunitx_unit_tmp_int=\count320
\l__siunitx_unit_position_int=\count321
\l__siunitx_unit_total_int=\count322
)
Package translations Info: No language package found. I am going to use
`englis
h' as default language. on input line 49.
LaTeX Font Info:    Trying to load font information for T1+Merriwthr-OsF
on inp
ut line 49.
(c:/TeXLive/2022/texmf-dist/tex/latex/merriweather/T1Merriwthr-OsF.fd
File: T1Merriwthr-OsF.fd 2020/08/30 (autoinst) Font definitions for
T1/Merriwth
r-OsF.
)
LaTeX Font Info:    Font shape `T1/Merriwthr-OsF/m/n' will be
(Font)              scaled to size 7.5pt on input line 49.
(./main.aux)
\openout1 = `main.aux'.

LaTeX Font Info:    Checking defaults for OML/cmm/m/it on input line 49.
LaTeX Font Info:    ... okay on input line 49.
LaTeX Font Info:    Checking defaults for OMS/cmsy/m/n on input line 49.
LaTeX Font Info:    ... okay on input line 49.
LaTeX Font Info:    Checking defaults for OT1/cmr/m/n on input line 49.
LaTeX Font Info:    ... okay on input line 49.
LaTeX Font Info:    Checking defaults for T1/cmr/m/n on input line 49.
LaTeX Font Info:    ... okay on input line 49.
LaTeX Font Info:    Checking defaults for TS1/cmr/m/n on input line 49.
LaTeX Font Info:    ... okay on input line 49.
LaTeX Font Info:    Checking defaults for OMX/cmex/m/n on input line 49.
LaTeX Font Info:    ... okay on input line 49.
LaTeX Font Info:    Checking defaults for U/cmr/m/n on input line 49.
LaTeX Font Info:    ... okay on input line 49.
LaTeX Font Info:    Checking defaults for PD1/pdf/m/n on input line 49.
LaTeX Font Info:    ... okay on input line 49.
LaTeX Font Info:    Checking defaults for PU/pdf/m/n on input line 49.
LaTeX Font Info:    ... okay on input line 49.
LaTeX Info: Redefining \microtypecontext on input line 49.
```

```
Package microtype Info: Applying patch `item' on input line 49.
Package microtype Info: Applying patch `toc' on input line 49.
Package microtype Info: Applying patch `eqnum' on input line 49.

Package microtype Warning: Unable to apply patch `footnote' on input line
49.

Package microtype Info: Applying patch `verbatim' on input line 49.
Package microtype Info: Generating PDF output.
Package microtype Info: Character protrusion enabled (level 2).
Package microtype Info: Using default protrusion set `alltext'.
Package microtype Info: Automatic font expansion enabled (level 2),
(microtype)             stretch: 20, shrink: 20, step: 1, non-selected.
Package microtype Info: Using default expansion set `alltext-nott'.
LaTeX Info: Redefining \showhyphens on input line 49.
Package microtype Info: No adjustment of tracking.
Package microtype Info: No adjustment of interword spacing.
Package microtype Info: No adjustment of character kerning.
Package microtype Info: Loading generic protrusion settings for font
family
(microtype)             `Merriwthr-OsF' (encoding: T1).
(microtype)             For optimal results, create family-specific
settings.
(microtype)             See the microtype manual for details.
LaTeX Font Info:    Redeclaring symbol font `operators' on input line 49.
LaTeX Font Info:    Encoding `OT1' has changed to `T1' for symbol font
(Font)              `operators' in the math version `normal' on input
line 49.
LaTeX Font Info:    Overwriting symbol font `operators' in version
`normal'
(Font)                  OT1/cmr/m/n --> T1/Merriwthr-OsF/m/up on input
line 49.

LaTeX Font Info:    Encoding `OT1' has changed to `T1' for symbol font
(Font)              `operators' in the math version `bold' on input line
49.
LaTeX Font Info:    Overwriting symbol font `operators' in version `bold'
(Font)                  OT1/cmr/bx/n --> T1/Merriwthr-OsF/m/up on input
line 49
.
LaTeX Font Info:    Overwriting symbol font `operators' in version `bold'
(Font)                  T1/Merriwthr-OsF/m/up --> T1/Merriwthr-OsF/b/up
on inpu
t line 49.
LaTeX Font Info:    Redeclaring math alphabet \mathbf on input line 49.
LaTeX Font Info:    Overwriting math alphabet `\mathbf' in version
`normal'
(Font)                  OT1/cmr/bx/n --> T1/Merriwthr-OsF/b/up on input
line 49
.
LaTeX Font Info:    Overwriting math alphabet `\mathbf' in version `bold'
(Font)                  OT1/cmr/bx/n --> T1/Merriwthr-OsF/b/up on input
line 49
.
```

```
LaTeX Font Info:    Redeclaring math alphabet \mathsf on input line 49.
LaTeX Font Info:    Overwriting math alphabet `\mathsf' in version
`normal'
(Font)                  OT1/cmss/m/n --> T1/MerriwthrSans-OsF/m/up on
input lin
e 49.
LaTeX Font Info:    Overwriting math alphabet `\mathsf' in version `bold'
(Font)                  OT1/cmss/bx/n --> T1/MerriwthrSans-OsF/m/up on
input li
ne 49.
LaTeX Font Info:    Redeclaring math alphabet \mathit on input line 49.
LaTeX Font Info:    Overwriting math alphabet `\mathit' in version
`normal'
(Font)                  OT1/cmr/m/it --> T1/Merriwthr-OsF/m/it on input
line 49
.
LaTeX Font Info:    Overwriting math alphabet `\mathit' in version `bold'
(Font)                  OT1/cmr/bx/it --> T1/Merriwthr-OsF/m/it on input
line 4
9.
LaTeX Font Info:    Redeclaring math alphabet \mathtt on input line 49.
LaTeX Font Info:    Overwriting math alphabet `\mathtt' in version
`normal'
(Font)                  OT1/cmtt/m/n --> T1/lmtt/m/up on input line 49.
LaTeX Font Info:    Overwriting math alphabet `\mathtt' in version `bold'
(Font)                  OT1/cmtt/m/n --> T1/lmtt/m/up on input line 49.
LaTeX Font Info:    Overwriting math alphabet `\mathsf' in version `bold'
(Font)                  T1/MerriwthrSans-OsF/m/up --> T1/MerriwthrSans-
OsF/b/up
 on input line 49.
LaTeX Font Info:    Overwriting math alphabet `\mathit' in version `bold'
(Font)                  T1/Merriwthr-OsF/m/it --> T1/Merriwthr-OsF/b/it
on inpu
t line 49.
\c@mv@tabular=\count323
\c@mv@boldtabular=\count324
(c:/TeXLive/2022/texmf-dist/tex/context/base/mkii/supp-pdf.mkii
[Loading MPS to PDF converter (version 2006.09.02).]
\scratchcounter=\count325
\scratchdimen=\dimen263
\scratchbox=\box75
\nofMPsegments=\count326
\nofMParguments=\count327
\everyMPshowfont=\toks39
\MPscratchCnt=\count328
\MPscratchDim=\dimen264
\MPnumerator=\count329
\makeMPintoPDFobject=\count330
\everyMPtoPDFconversion=\toks40
) (c:/TeXLive/2022/texmf-dist/tex/latex/epstopdf-pkg/epstopdf-base.sty
Package: epstopdf-base 2020-01-24 v2.11 Base part for package epstopdf
Package epstopdf-base Info: Redefining graphics rule for `.eps' on input
line 4
85.
```

```
(c:/TeXLive/2022/texmf-dist/tex/latex/latexconfig/epstopdf-sys.cfg
File: epstopdf-sys.cfg 2010/07/13 v1.3 Configuration of (r)epstopdf for
TeX Liv
e
))
*geometry* driver: auto-detecting
*geometry* detected driver: pdftex
*geometry* verbose mode - [ preamble ] result:
* driver: pdftex
* paper: a4paper
* layout: <same size as paper>
* layoutoffset:(h,v)=(0.0pt,0.0pt)
* modes: includefoot twoside
* h-part:(L,W,R)=(54.64pt, 488.22787pt, 54.64pt)
* v-part:(T,H,B)=(66.0pt, 745.04684pt, 34.0pt)
* \paperwidth=597.50787pt
* \paperheight=845.04684pt
* \textwidth=488.22787pt
* \textheight=715.04684pt
* \oddsidemargin=-17.62999pt
* \evensidemargin=-17.62999pt
* \topmargin=-47.76999pt
* \headheight=17.5pt
* \headsep=24.0pt
* \topskip=10.0pt
* \footskip=30.0pt
* \marginparwidth=48.0pt
* \marginparsep=10.0pt
* \columnsep=18.0pt
* \skip\footins=22.0pt plus 2.0pt
* \hoffset=0.0pt
* \voffset=0.0pt
* \mag=1000
* \@twocolumntrue
* \@twosidetrue
* \@mparswitchtrue
* \@reversemarginfalse
* (1in=72.27pt=25.4mm, 1cm=28.453pt)

Package hyperref Info: Link coloring ON on input line 49.
(./main.out) (./main.out)
\@outlinefile=\write3
\openout3 = `main.out'.

\@gscitedetails=\box76
\@gscitedetailsheight=\skip163
\@gsheadbox=\box77
\@gsheadboxheight=\skip164
LaTeX Font Info:    Font shape `T1/Merriwthr-OsF/b/n' will be
(Font)              scaled to size 6.5pt on input line 49.
LaTeX Font Info:    Calculating math sizes for size <7.5> on input line
49.

LaTeX Font Warning: Font shape `T1/Merriwthr-OsF/m/up' undefined
```

```
(Font)              using `T1/Merriwthr-OsF/m/n' instead on input line
49.

LaTeX Font Info:    Font shape `T1/Merriwthr-OsF/m/up' will be
(Font)              scaled to size 6.24973pt on input line 49.
LaTeX Font Info:    Font shape `T1/Merriwthr-OsF/m/up' will be
(Font)              scaled to size 5.24997pt on input line 49.
LaTeX Font Info:    Trying to load font information for U+eur on input
line 49.

(c:/TeXLive/2022/texmf-dist/tex/latex/amsfonts/ueur.fd
File: ueur.fd 2013/01/14 v3.01 Euler Roman
) (c:/TeXLive/2022/texmf-dist/tex/latex/microtype/mt-eur.cfg
File: mt-eur.cfg 2006/07/31 v1.1 microtype config. file: AMS Euler Roman
(RS)
)

LaTeX Font Warning: Font shape `OMS/cmsy/m/n' in size <7.5> not available
(Font)              size <7> substituted on input line 49.

LaTeX Font Info:    External font `cmex10' loaded for size
(Font)              <7.5> on input line 49.
LaTeX Font Info:    External font `cmex10' loaded for size
(Font)              <6.24973> on input line 49.
LaTeX Font Info:    External font `cmex10' loaded for size
(Font)              <5.24997> on input line 49.
LaTeX Font Info:    Trying to load font information for U+euf on input
line 49.

(c:/TeXLive/2022/texmf-dist/tex/latex/amsfonts/ueuf.fd
File: ueuf.fd 2013/01/14 v3.01 Euler Fraktur
) (c:/TeXLive/2022/texmf-dist/tex/latex/microtype/mt-euf.cfg
File: mt-euf.cfg 2006/07/03 v1.1 microtype config. file: AMS Euler
Fraktur (RS)

)
LaTeX Font Info:    Trying to load font information for U+eus on input
line 49.

(c:/TeXLive/2022/texmf-dist/tex/latex/amsfonts/ueus.fd
File: ueus.fd 2013/01/14 v3.01 Euler Script
) (c:/TeXLive/2022/texmf-dist/tex/latex/microtype/mt-eus.cfg
File: mt-eus.cfg 2006/07/28 v1.2 microtype config. file: AMS Euler Script
(RS)
)
LaTeX Font Info:    Trying to load font information for U+euex on input
line 49
.
(c:/TeXLive/2022/texmf-dist/tex/latex/amsfonts/ueuex.fd
File: ueuex.fd 2013/01/14 v3.01 Euler extra symbols
)

LaTeX Font Warning: Font shape `OML/cmm/m/it' in size <7.5> not available
(Font)              size <7> substituted on input line 49.
```

```
LaTeX Font Info:    Font shape `T1/Merriwthr-OsF/m/n' will be
(Font)              scaled to size 6.24973pt on input line 49.
LaTeX Font Info:    Font shape `T1/Merriwthr-OsF/m/n' will be
(Font)              scaled to size 5.24997pt on input line 49.
LaTeX Font Info:    Font shape `T1/Merriwthr-OsF/m/it' will be
(Font)              scaled to size 7.5pt on input line 49.
LaTeX Font Info:    Font shape `T1/Merriwthr-OsF/m/it' will be
(Font)              scaled to size 6.24973pt on input line 49.
LaTeX Font Info:    Font shape `T1/Merriwthr-OsF/m/it' will be
(Font)              scaled to size 5.24997pt on input line 49.
LaTeX Font Info:    Font shape `T1/Merriwthr-OsF/m/n' will be
(Font)              scaled to size 8.0pt on input line 49.
LaTeX Font Info:    Font shape `T1/Merriwthr-OsF/m/it' will be
(Font)              scaled to size 8.0pt on input line 49.
LaTeX Font Info:    Font shape `T1/Merriwthr-OsF/b/it' will be
(Font)              scaled to size 8.0pt on input line 49.
Package caption Info: Begin \AtBeginDocument code.
Package caption Info: End \AtBeginDocument code.

(c:/TeXLive/2022/texmf-dist/tex/latex/translations/translations-basic-
dictionar
y-english.trsl
File: translations-basic-dictionary-english.trsl (english translation
file `tra
nslations-basic-dictionary')
)
Package translations Info: loading dictionary `translations-basic-
dictionary' f
or `english'. on input line 49.
TextBlockOrigin set to 4pc+6.64pt x 4pc+6pt
<oup.pdf, id=117, 49.18375pt x 48.18pt>
File: oup.pdf Graphic file (type pdf)
<use oup.pdf>
Package pdftex.def Info: oup.pdf  used on input line 70.
(pdftex.def)             Requested size: 59.24683pt x 58.038pt.
<gigascience-logo.pdf, id=118, 99.37125pt x 33.12375pt>
File: gigascience-logo.pdf Graphic file (type pdf)
<use gigascience-logo.pdf>
Package pdftex.def Info: gigascience-logo.pdf  used on input line 70.
(pdftex.def)             Requested size: 126.00902pt x 42.0pt.

Overfull \hbox (54.64pt too wide) in paragraph at lines 70--70
[][]
 []

LaTeX Font Info:    Font shape `T1/Merriwthr-OsF/m/n' will be
(Font)              scaled to size 14.0pt on input line 70.
LaTeX Font Info:    Font shape `T1/Merriwthr-OsF/m/n' will be
(Font)              scaled to size 8.99997pt on input line 70.
LaTeX Font Info:    Calculating math sizes for size <14> on input line
70.
LaTeX Font Info:    Font shape `T1/Merriwthr-OsF/m/up' will be
(Font)              scaled to size 14.0pt on input line 70.
```

```
LaTeX Font Info:    Font shape `T1/Merriwthr-OsF/m/up' will be
(Font)              scaled to size 11.66617pt on input line 70.
LaTeX Font Info:    Font shape `T1/Merriwthr-OsF/m/up' will be
(Font)              scaled to size 9.79996pt on input line 70.
LaTeX Font Info:    External font `cmex10' loaded for size
(Font)              <14> on input line 70.
LaTeX Font Info:    External font `cmex10' loaded for size
(Font)              <11.66617> on input line 70.
LaTeX Font Info:    External font `cmex10' loaded for size
(Font)              <9.79996> on input line 70.
LaTeX Font Info:    Font shape `T1/Merriwthr-OsF/m/n' will be
(Font)              scaled to size 11.66617pt on input line 70.
LaTeX Font Info:    Font shape `T1/Merriwthr-OsF/m/n' will be
(Font)              scaled to size 9.79996pt on input line 70.
LaTeX Font Info:    Font shape `T1/Merriwthr-OsF/m/it' will be
(Font)              scaled to size 14.0pt on input line 70.
LaTeX Font Info:    Font shape `T1/Merriwthr-OsF/m/it' will be
(Font)              scaled to size 11.66617pt on input line 70.
LaTeX Font Info:    Font shape `T1/Merriwthr-OsF/m/it' will be
(Font)              scaled to size 9.79996pt on input line 70.
LaTeX Font Info:    Font shape `T1/Merriwthr-OsF/b/n' will be
(Font)              scaled to size 18.0pt on input line 70.
LaTeX Font Info:    Font shape `T1/Merriwthr-OsF/m/n' will be
(Font)              scaled to size 13.0pt on input line 70.
LaTeX Font Info:    Calculating math sizes for size <13> on input line
70.
LaTeX Font Info:    Font shape `T1/Merriwthr-OsF/m/up' will be
(Font)              scaled to size 13.0pt on input line 70.
LaTeX Font Info:    Font shape `T1/Merriwthr-OsF/m/up' will be
(Font)              scaled to size 10.83287pt on input line 70.
LaTeX Font Info:    Font shape `T1/Merriwthr-OsF/m/up' will be
(Font)              scaled to size 9.09996pt on input line 70.

LaTeX Font Warning: Font shape `OMS/cmsy/m/n' in size <13> not available
(Font)              size <12> substituted on input line 70.

LaTeX Font Info:    External font `cmex10' loaded for size
(Font)              <13> on input line 70.
LaTeX Font Info:    External font `cmex10' loaded for size
(Font)              <10.83287> on input line 70.
LaTeX Font Info:    External font `cmex10' loaded for size
(Font)              <9.09996> on input line 70.

LaTeX Font Warning: Font shape `OML/cmm/m/it' in size <13> not available
(Font)              size <12> substituted on input line 70.

LaTeX Font Info:    Font shape `T1/Merriwthr-OsF/m/n' will be
(Font)              scaled to size 10.83287pt on input line 70.
LaTeX Font Info:    Font shape `T1/Merriwthr-OsF/m/n' will be
(Font)              scaled to size 9.09996pt on input line 70.
LaTeX Font Info:    Font shape `T1/Merriwthr-OsF/m/it' will be
(Font)              scaled to size 13.0pt on input line 70.
LaTeX Font Info:    Font shape `T1/Merriwthr-OsF/m/it' will be
(Font)              scaled to size 10.83287pt on input line 70.
```

```
LaTeX Font Info:    Font shape `T1/Merriwthr-OsF/m/it' will be
(Font)              scaled to size 9.09996pt on input line 70.
LaTeX Font Info:    Trying to load font information for TS1+Merriwthr-OsF
on in
put line 70.
(c:/TeXLive/2022/texmf-dist/tex/latex/merriweather/TS1Merriwthr-OsF.fd
File: TS1Merriwthr-OsF.fd 2020/08/30 (autoinst) Font definitions for
TS1/Merriw
thr-OsF.
)
LaTeX Font Info:    Font shape `TS1/Merriwthr-OsF/m/n' will be
(Font)              scaled to size 10.83287pt on input line 70.
Package microtype Info: Loading generic protrusion settings for font
family
(microtype)             `Merriwthr-OsF' (encoding: TS1).
(microtype)             For optimal results, create family-specific
settings.
(microtype)             See the microtype manual for details.
LaTeX Font Info:    Font shape `T1/Merriwthr-OsF/m/n' will be
(Font)              scaled to size 9.0pt on input line 70.
LaTeX Font Info:    Font shape `T1/Merriwthr-OsF/m/up' will be
(Font)              scaled to size 9.0pt on input line 70.
LaTeX Font Info:    Font shape `T1/Merriwthr-OsF/m/up' will be
(Font)              scaled to size 7.0pt on input line 70.
LaTeX Font Info:    Font shape `T1/Merriwthr-OsF/m/up' will be
(Font)              scaled to size 5.0pt on input line 70.
LaTeX Font Info:    External font `cmex10' loaded for size
(Font)              <9> on input line 70.
LaTeX Font Info:    External font `cmex10' loaded for size
(Font)              <7> on input line 70.
LaTeX Font Info:    External font `cmex10' loaded for size
(Font)              <5> on input line 70.
LaTeX Font Info:    Font shape `T1/Merriwthr-OsF/m/n' will be
(Font)              scaled to size 7.0pt on input line 70.
LaTeX Font Info:    Font shape `T1/Merriwthr-OsF/m/n' will be
(Font)              scaled to size 5.0pt on input line 70.
LaTeX Font Info:    Font shape `T1/Merriwthr-OsF/m/it' will be
(Font)              scaled to size 9.0pt on input line 70.
LaTeX Font Info:    Font shape `T1/Merriwthr-OsF/m/it' will be
(Font)              scaled to size 7.0pt on input line 70.
LaTeX Font Info:    Font shape `T1/Merriwthr-OsF/m/it' will be
(Font)              scaled to size 5.0pt on input line 70.
LaTeX Font Info:    Font shape `T1/Merriwthr-OsF/m/n' will be
(Font)              scaled to size 6.5pt on input line 70.
LaTeX Font Info:    Calculating math sizes for size <6.5> on input line
70.
LaTeX Font Info:    Font shape `T1/Merriwthr-OsF/m/up' will be
(Font)              scaled to size 6.5pt on input line 70.
LaTeX Font Info:    Font shape `T1/Merriwthr-OsF/m/up' will be
(Font)              scaled to size 5.41643pt on input line 70.
LaTeX Font Info:    Font shape `T1/Merriwthr-OsF/m/up' will be
(Font)              scaled to size 4.54997pt on input line 70.

LaTeX Font Warning: Font shape `OMS/cmsy/m/n' in size <6.5> not available
```

```
(Font)              size <6> substituted on input line 70.


LaTeX Font Warning: Font shape `OMS/cmsy/m/n' in size <5.41643> not
available
(Font)              size <5> substituted on input line 70.


LaTeX Font Warning: Font shape `OMS/cmsy/m/n' in size <4.54997> not
available
(Font)              size <5> substituted on input line 70.

LaTeX Font Info:    External font `cmex10' loaded for size
(Font)              <6.5> on input line 70.
LaTeX Font Info:    External font `cmex10' loaded for size
(Font)              <5.41643> on input line 70.
LaTeX Font Info:    External font `cmex10' loaded for size
(Font)              <4.54997> on input line 70.

LaTeX Font Warning: Font shape `OML/cmm/m/it' in size <6.5> not available
(Font)              size <6> substituted on input line 70.


LaTeX Font Warning: Font shape `OML/cmm/m/it' in size <5.41643> not
available
(Font)              size <5> substituted on input line 70.


LaTeX Font Warning: Font shape `OML/cmm/m/it' in size <4.54997> not
available
(Font)              size <5> substituted on input line 70.

LaTeX Font Info:    Font shape `T1/Merriwthr-OsF/m/n' will be
(Font)              scaled to size 5.41643pt on input line 70.
LaTeX Font Info:    Font shape `T1/Merriwthr-OsF/m/n' will be
(Font)              scaled to size 4.54997pt on input line 70.
LaTeX Font Info:    Font shape `T1/Merriwthr-OsF/m/it' will be
(Font)              scaled to size 6.5pt on input line 70.
LaTeX Font Info:    Font shape `T1/Merriwthr-OsF/m/it' will be
(Font)              scaled to size 5.41643pt on input line 70.
LaTeX Font Info:    Font shape `T1/Merriwthr-OsF/m/it' will be
(Font)              scaled to size 4.54997pt on input line 70.
LaTeX Font Info:    Font shape `TS1/Merriwthr-OsF/m/n' will be
(Font)              scaled to size 5.41643pt on input line 70.

Overfull \hbox (54.64pt too wide) in paragraph at lines 70--70
[][][]
 []

LaTeX Font Info:    Font shape `T1/Merriwthr-OsF/b/n' will be
(Font)              scaled to size 10.0pt on input line 70.
LaTeX Font Info:    Font shape `T1/Merriwthr-OsF/b/n' will be
(Font)              scaled to size 8.0pt on input line 70.
```

```
Overfull \hbox (54.64pt too wide) in paragraph at lines 70--70
[][][]
 []

Package mdframed Info: mdframed works in twoside mode on input line 73.
LaTeX Font Info:    Font shape `T1/Merriwthr-OsF/b/n' will be
(Font)              scaled to size 8.2pt on input line 73.
LaTeX Font Info:    Font shape `TS1/Merriwthr-OsF/m/n' will be
(Font)              scaled to size 7.5pt on input line 75.
Package mdframed Info: mdframed inside float
 mdframed uses option nobreak mdframed on input line 80.
Package mdframed Info: mdframed inside a box
 mdframed uses option nobreak mdframed on input line 80.
LaTeX Font Info:    Font shape `T1/Merriwthr-OsF/b/n' will be
(Font)              scaled to size 8.5pt on input line 85.

Underfull \vbox (badness 10000) has occurred while \output is active []

LaTeX Font Info:    Font shape `T1/Merriwthr-OsF/m/n' will be
(Font)              scaled to size 7.8pt on input line 91.
LaTeX Font Info:    Font shape `T1/Merriwthr-OsF/b/n' will be
(Font)              scaled to size 7.8pt on input line 91.
[1{c:/TeXLive/2022/texmf-var/fonts/map/pdftex/updmap/pdftex.map}


 <./oup.pdf> <./gigascience-logo.pdf>]
LaTeX Font Info:    Font shape `T1/Merriwthr-OsF/b/n' will be
(Font)              scaled to size 7.5pt on input line 98.
LaTeX Font Info:    Font shape `T1/Merriwthr-OsF/b/sl' in size <7.5> not
availa
ble
(Font)              Font shape `T1/Merriwthr-OsF/b/it' tried instead on
input l
ine 98.
LaTeX Font Info:    Font shape `T1/Merriwthr-OsF/b/it' will be
(Font)              scaled to size 7.5pt on input line 98.
LaTeX Font Info:    Font shape `T1/Merriwthr-OsF/m/up' will be
(Font)              scaled to size 7.5pt on input line 101.
LaTeX Font Info:    Font shape `T1/Merriwthr-OsF/m/n' will be
(Font)              scaled to size 6.25008pt on input line 101.
LaTeX Font Info:    Trying to load font information for T1+lmtt on input
line 1
01.
(c:/TeXLive/2022/texmf-dist/tex/latex/lm/t1lmtt.fd
File: t1lmtt.fd 2015/05/01 v1.6.1 Font defs for Latin Modern
)
Package microtype Info: Loading generic protrusion settings for font
family
(microtype)             `lmtt' (encoding: T1).
(microtype)             For optimal results, create family-specific
settings.
(microtype)             See the microtype manual for details.
LaTeX Font Info:    Calculating math sizes for size <6.25008> on input
line 101
```

```
.
LaTeX Font Info:    Font shape `T1/Merriwthr-OsF/m/up' will be
(Font)              scaled to size 6.25008pt on input line 101.
LaTeX Font Info:    Font shape `T1/Merriwthr-OsF/m/up' will be
(Font)              scaled to size 5.20816pt on input line 101.
LaTeX Font Info:    Font shape `T1/Merriwthr-OsF/m/up' will be
(Font)              scaled to size 4.37503pt on input line 101.

LaTeX Font Warning: Font shape `OMS/cmsy/m/n' in size <4.37503> not
available
(Font)              size <5> substituted on input line 101.

LaTeX Font Info:    External font `cmex10' loaded for size
(Font)              <6.25008> on input line 101.
LaTeX Font Info:    External font `cmex10' loaded for size
(Font)              <5.20816> on input line 101.
LaTeX Font Info:    External font `cmex10' loaded for size
(Font)              <4.37503> on input line 101.

LaTeX Font Warning: Font shape `OML/cmm/m/it' in size <4.37503> not
available
(Font)              size <5> substituted on input line 101.

LaTeX Font Info:    Font shape `T1/Merriwthr-OsF/m/n' will be
(Font)              scaled to size 5.20816pt on input line 101.
LaTeX Font Info:    Font shape `T1/Merriwthr-OsF/m/n' will be
(Font)              scaled to size 4.37503pt on input line 101.
LaTeX Font Info:    Font shape `T1/Merriwthr-OsF/m/it' will be
(Font)              scaled to size 6.25008pt on input line 101.
LaTeX Font Info:    Font shape `T1/Merriwthr-OsF/m/it' will be
(Font)              scaled to size 5.20816pt on input line 101.
LaTeX Font Info:    Font shape `T1/Merriwthr-OsF/m/it' will be
(Font)              scaled to size 4.37503pt on input line 101.

Underfull \hbox (badness 10000) in paragraph at lines 101--101
 [][]\T1/Merriwthr-OsF/m/up/6.25008 (+20) Possible via
[][]$\T1/lmtt/m/n/6.2500
8 https : / / ega . ebi . ac . uk : 8443 / ega-[]openid-[]connect-
[]server /
 []


! LaTeX Error: File `figures/figure1' not found.

See the LaTeX manual or LaTeX Companion for explanation.
Type  H <return>  for immediate help.
 ...

l.108 ...aphics[width=\linewidth]{figures/figure1}

I could not locate the file with any of these extensions:
.pdf,.png,.jpg,.mps,.jpeg,.jbig2,.jb2,.PDF,.PNG,.JPG,.JPEG,.JBIG2,.JB2,.e
ps
Try typing  <return>  to proceed.
```

```
If that doesn't work, type  X <return>  to quit.

LaTeX Font Info:    Font shape `T1/Merriwthr-OsF/m/n' will be
(Font)              scaled to size 6.0pt on input line 109.
LaTeX Font Info:    Font shape `T1/Merriwthr-OsF/b/n' will be
(Font)              scaled to size 6.0pt on input line 109.
LaTeX Font Info:    Font shape `T1/Merriwthr-OsF/m/it' will be
(Font)              scaled to size 7.8pt on input line 120.
[2]

LaTeX Warning: File `figures/figure2.pdf' not found on input line 132.


! Package pdftex.def Error: File `figures/figure2.pdf' not found: using
draft s
etting.

See the pdftex.def package documentation for explanation.
Type  H <return>  for immediate help.
 ...

l.132 ...cs[width=\linewidth]{figures/figure2.pdf}

Try typing  <return>  to proceed.
If that doesn't work, type  X <return>  to quit.

LaTeX Font Info:    Font shape `T1/Merriwthr-OsF/b/n' will be
(Font)              scaled to size 7.0pt on input line 143.
[3]
Underfull \vbox (badness 7308) has occurred while \output is active []


Underfull \vbox (badness 10000) has occurred while \output is active []

[4]
Underfull \hbox (badness 10000) in paragraph at lines 199--200
[]\T1/Merriwthr-OsF/m/up/7.5 (+20) Github repos-i-tory:
[][]$\T1/lmtt/m/n/7.5 h
ttps : / / github . com / galaxyproject /
 []


Underfull \hbox (badness 10000) in paragraph at lines 201--202
[]\T1/Merriwthr-OsF/m/up/7.5 (+20) Training Man-ual:
[][]$\T1/lmtt/m/n/7.5 http
s : / / training . galaxyproject . org /
 []


Underfull \hbox (badness 10000) in paragraph at lines 201--202
\T1/lmtt/m/n/7.5 training-[]material / topics / variant-[]analysis /
tutorials
/
 []
```

```
Underfull \hbox (badness 10000) in paragraph at lines 210--211
[]\T1/Merriwthr-OsF/m/up/7.5 (+20) Github repos-i-tory:
[][]$\T1/lmtt/m/n/7.5 h
ttps : / / github . com / galaxyproject /
 []


Underfull \hbox (badness 10000) in paragraph at lines 212--213
[]\T1/Merriwthr-OsF/m/up/7.5 (+20) Training Man-ual:
[][]$\T1/lmtt/m/n/7.5 http
s : / / training . galaxyproject . org /
 []


Underfull \hbox (badness 10000) in paragraph at lines 212--213
\T1/lmtt/m/n/7.5 training-[]material / topics / variant-[]analysis /
tutorials
/
 []

(./main.bbl
Underfull \hbox (badness 10000) in paragraph at lines 88--91
[]\T1/Merriwthr-OsF/m/up/7.5 (+20) Galaxy Train-ing Net-work Stats;.
[][]$\T1/
lmtt/m/n/7.5 https : / / training .
 []

[5]
Underfull \hbox (badness 10000) in paragraph at lines 170--173
[]\T1/Merriwthr-OsF/m/up/7.5 (+20) Galaxy Train-ing Net-work Stats;.
[][]$\T1/
lmtt/m/n/7.5 https : / / training .
 []


Underfull \hbox (badness 10000) in paragraph at lines 175--179
[][]$\T1/lmtt/m/n/7.5 https : / / training . galaxyproject . org /
training-[]m
aterial /
 []


Underfull \hbox (badness 10000) in paragraph at lines 175--179
\T1/lmtt/m/n/7.5 topics / variant-[]analysis / tutorials / trio-
[]analysis /
 []


Underfull \hbox (badness 10000) in paragraph at lines 223--226
[]\T1/Merriwthr-OsF/m/up/7.5 (+20) HapMap Project;.
[][]$\T1/lmtt/m/n/7.5 www
. genome . gov / 10001688 /
```

```
 []


Underfull \hbox (badness 4181) in paragraph at lines 232--235
[]\T1/Merriwthr-OsF/m/up/7.5 (+20) CINECA Project;.
[][]$\T1/lmtt/m/n/7.5 http
s : / / cordis . europa . eu / project / id /
 []


Underfull \hbox (badness 3919) in paragraph at lines 242--245
[]\T1/Merriwthr-OsF/m/up/7.5 (+20) EJP-RD Project;.
[][]$\T1/lmtt/m/n/7.5 http
s : / / cordis . europa . eu / project / id /
 []


Underfull \hbox (badness 10000) in paragraph at lines 247--250
[]\T1/Merriwthr-OsF/m/up/7.5 (+20) B1MG Project;.  [][]$\T1/lmtt/m/n/7.5
https
: / / cordis . europa . eu / project / id /
 []


Overfull \hbox (291.31828pt too wide) in paragraph at lines 252--256
\T1/lmtt/m/n/7.5 projects / 25 / veis-[]european-[]project-[]valuation-
[]of-[]t
he-[]european-[]archive-[]of-[]the-[]genome-[]phenome-[]ega-[]for-[]the-
[]indus
try-[]and-[]the-[]society$[][]\T1/Merriwthr-OsF/m/up/7.5 (-20) ,
 []

) [6

]
enddocument/afterlastpage: lastpage setting LastPage.
(./main.aux)

LaTeX Font Warning: Size substitutions with differences
(Font)              up to 1.0pt have occurred.


LaTeX Font Warning: Some font shapes were not available, defaults
substituted.

Package rerunfilecheck Info: File `main.out' has not changed.
(rerunfilecheck)             Checksum:
D45578C491808653652FDBFB3CF5EB77;3874.
 )
Here is how much of TeX's memory you used:
 24039 strings out of 476024
 468724 string characters out of 5794017
 1897382 words of memory out of 5000000
 43505 multiletter control sequences out of 15000+600000
```

```
 1921786 words of font info for 488 fonts, out of 8000000 for 9000
 1141 hyphenation exceptions out of 8191
 123i,12n,131p,1345b,1065s stack positions out of
10000i,1000n,20000p,200000b,200000s

pdfTeX warning (dest): name{Hfootnote.2} has been referenced but does not
exist
, replaced by a fixed one


pdfTeX warning (dest): name{Hfootnote.1} has been referenced but does not
exist
, replaced by a fixed one

{c:/TeXLive/2022/texmf-dist/fonts/enc/dvips/lm/lm-
ec.enc}{c:/TeXLive/2022/texmf
-
dist/fonts/enc/dvips/merriweather/merriwthr_posqbl.enc}{c:/TeXLive/2022/t
exmf-
dist/fonts/enc/dvips/merriweather/merriwthr_owzwzj.enc}<c:/TeXLive/2022/t
exmf-d
ist/fonts/type1/sorkin/merriweather/Merriwthr-
Bold.pfb><c:/TeXLive/2022/texmf-d
ist/fonts/type1/sorkin/merriweather/Merriwthr-
BoldItalic.pfb><c:/TeXLive/2022/t
exmf-dist/fonts/type1/sorkin/merriweather/Merriwthr-
Italic.pfb><c:/TeXLive/2022
/texmf-dist/fonts/type1/sorkin/merriweather/Merriwthr-
Regular.pfb><c:/TeXLive/2
022/texmf-dist/fonts/type1/public/lm/lmtt8.pfb>
Output written on main.pdf (6 pages, 330189 bytes).
PDF statistics:
 341 PDF objects out of 1000 (max. 8388607)
 314 compressed objects within 4 object streams
 73 named destinations out of 1000 (max. 500000)
 214251 words of extra memory for PDF output out of 221844 (max.
10000000)
```

Manuscript
Click here to access/download;Manuscript;main.pdf

OXFORD
(GIGA)n SCIENCE

GigaScience, 2017, 1–6

doi: xx.xxxx/xxxx
Manuscript in Preparation
Paper

PAPER

FAIR Data Retrieval for Sensitive Clinical Analysis in Galaxy

Jasper Ouwerkerk[1,*,†], Helena Rasche[1,†], John D. Spalding[2], Saskia Hiltemann[1,††] and Andrew P. Stubbs[1,††]

[1]Erasmus Medical Center, Clinical Bioinformatics Group, Department of Pathology, Wytemaweg 80, 3015 CN, Rotterdam, The Netherlands and [2]CSC–IT Center for Science, Keilaranta 14, 02101, Espoo, Finland

[*]Correspondence address. Jasper Ouwerkerk, Clinical Bioinformatics Group, Department of Pathology, Erasmus Medical Center, Wytemaweg 80, 3015 CN, Rotterdam, The Netherlands. E-mail: j.ouwerkerk.1@erasmusmc.nl
[†]Contributed equally.

Abstract

Background, In clinical research, data has to be accessible and reproducible, but the generated data is becoming larger and analysis complex. Here we propose a platform for FAIR data access and creating reproducible findings. Standardised access to a major genomic repository, the European Genome-Phenome Archive (EGA), has been achieved with API services like PyEGA3. We aim to provide a FAIR data analysis service in Galaxy by retrieving genomic data from the EGA and provide a generalised "omics" platform for FAIR data analysis.
Results, To demonstrate this, we implemented an end-to-end Galaxy workflow to replicate the findings from an RD-Connect synthetic dataset Beyond the 1 Million Genomes (synB1MG) available from the EGA. We developed the PyEGA3 connector within Galaxy to easily download multiple datasets from the EGA. We added the gene.iobio tool, a diagnostic environment for precision genomics, to Galaxy and demonstrate that it provides a more dynamic and interpretable view for trio analysis results. We developed a Galaxy trio analysis workflow to determine the pathogenic variants from the synB1MG trios using the GEMINI and gene.iobio tool. The complete workflow is available at WorkflowHub and an associated tutorial was created in the Galaxy Training Network which helps researchers unfamiliar with Galaxy to run the workflow.
Conclusions, We showed the feasibility of reusing data from the EGA in Galaxy via PyEGA3 and validated the workflow by re-discovering spiked-in variants in synthetic data. Finally, we improved existing tools in Galaxy and created a workflow for trio analysis to demonstrate the value of FAIR genomics analysis in Galaxy.

Key words: B1MG; FAIR; Galaxy; Trio Analysis

Findings

Background

In the last few years there have been many developments in Findable, Accessible, Interoperable, and Reusable (FAIR) data [1]. FAIR data is data and corresponding metadata which are 1) findable by both machines and humans, 2) accessible using a standard open protocol, 3) interoperable so it can easily be processed and analysed, 4) resuable so the data can be understood by anyone and make analyses reproducible [2]. FAIR data allows researchers to reanalyse data with new genetic analysis tools not yet available at the time of data publication. For example, in a study on fusion genes, 24 novel fusion in breast cancer were found with the introduction of a new tool [3].

However, for many biomedical analyses, researchers are required to have considerable knowledge on using analysis tools. Moreover, many tools require knowledge of Unix commands or python coding. [4, 5, 6]. This creates a barrier for clinical researchers that want to reanalyse data, reducing the adoption of implementing FAIR principles and reanalyses.

Compiled on: June 28, 2023.
Draft manuscript prepared by the author.

Key Points

- Secure access to GA4GH EGA service using PyEGA3 Galaxy service
- Standard analysis for B1MG synthetic data
- Interactive gene variant detection for trio analysis with gene.iobio in Galaxy
- Created a tutorial associated with the Galaxy Training Network

The Galaxy platform [7] supports researchers in adopting these complex computation tools for their FAIR data analysis. Galaxy is an online analysis platform with a plethora of tools to perform text/table processing, omics analysis, machine learning, image analysis, and more. All these tools are maintained and developed by a growing community. Using the tools does not require any programming skills and are easy to share with colleagues and other researchers. At the end of the analysis the workflow of tools can be exported to reproduce the analysis [7]. These workflows can be made discoverable by uploading the workflow to WorkflowHub [8], a registry for describing, sharing, and publishing scientific computational workflows. In addition, Galaxy already has 300+ tutorials describing workflows on genome assembly, ecology, metagenomics, variant analysis and more [9]. This is beneficial to many researchers since there are many complex Unix-based tools which are essential for biomedical research. An example of such an application is Circos [6], which is a complicated visualization tool for comparing whole genomes. This tool has been implemented within Galaxy, which makes it simple for any researcher to create Circos plots [10].

Even though Galaxy is a great platform for analysis, it still lacks applications for retrieving access-controlled data from large repositories like the EGA. The EGA controls the accessibility to datasets using Data Access Committees (DACs). Requestors can access data from the EGA by contacting the DAC for the dataset of interest. DACs are generally formed by the organization which collected the data and performed the analysis. This allows researchers to access datasets of interest and also manage the accessibility of their data at the EGA [11].

In this work we implemented PyEGA3[12], a tool which can access controlled data from the EGA, within Galaxy. Here access to datasets is managed via the EGA. Our implementation of the PyEGA3 tool allows to filter datasets, available on the EGA, based on their metadata and scale up analysis. This will be showcased by validating our workflow for trio analysis on family trios from the Beyond 1 Million Genomes (B1MG) project [13]. Moreover, we added the gene.iobio tool for variant analysis, including trio analysis [14]. The complete workflow, including data retrieval with PyEGA3, is implemented within Galaxy and uploaded to WorkflowHub for discoverability. In addition, we wrote a tutorial to explain our workflow in detail, which is associated with the Galaxy Training Network (GTN) [15]. This study shows it is feasible to adopt end-to-end scalable FAIR analysis of clinical data, and ultimately for any future analysis on data available at the EGA.

Results

PyEGA3

PyEGA3 was implemented to retrieve access controlled data from the EGA in Galaxy. Authentication of the user is either done by password and username or Authentication and Authorization Infrastructure (AAI) tokens. Currently, the process is initiated by linking one's EGA account to their ELIXIR AAI[1] (now LS Login) account. Next, the user logs in to Galaxy via LS Login, which attaches the user's GA4GH passport and access and refresh tokens to the user's account in Galaxy. The refresh token is used to regularly refresh their credentials allowing the Galaxy server to act on their behalf when the user requests it via tool execution. Upon executing a tool, assuming the tool is written to support it, the access token, or possibly in the future passports, are attached to the tool's execution such that they can be used to authenticate the user. While currently the access token is implemented on an ad-hoc basis, we intend to directly implement support for this type of tool and authentication method in a future version of Galaxy[2] The tool implemented in Galaxy has the same functionalities as the command line version, namely list a user's authorized datasets, list files in a dataset and fetch a file or all files in a dataset. In addition, we added the option to download a specified list of files from the EGA. With this option it is still possible to download a specific genomic range, see Figure 1, which is useful for large binary alignment map (BAM) and variant calling format (VCF) files.

Figure 1. The Galaxy interface of the added feature to the PyEga3 tool to download multiple files. It takes a tabular data with EGAF IDs. In addition, a region can be provided to download a small region in BAMs or VCFs.

Gene.iobio

We also implemented the gene.iobio tool within the Galaxy framework. Gene.iobio is a tool for precision genomics. The tool is able to create dynamic results, which include creating a list of genes for the disease of interest, creating an automatic report of pathogenic variants within the list of genes, allowing the custom filtering of pathogenic variants, reporting phenotypes and publications related to the gene of interest, and reviewing the variants. These are major improvements compared to the existing trio analysis tool within Galaxy, GEMINI [16], which was only able to produce static plots or large lists of filtered variants.

Workflow & Tutorial

In this study we illustrate an end-to-end workflow for trio analysis for FAIR data. This workflow retrieves and analyses files from large datasets in the EGA and can easily be adapted to any other EGA dataset. We illustrate this by analysing data from the EGAD00001008392 [17] dataset. This dataset contains 6 trio families with different inheritance patterns of digitally spiked-in variants, where each family is subject to a different disease. Next, we demonstrate the usefulness of gene.iobio by analysing the family trios and comparing the existing trio analysis tool in Galaxy, GEMINI, to the gene.iobio tool. Finally, a comprehensive tutorial is made available at the Galaxy Training materials [18] under the topic 'Variant Analysis' titled 'Trio Analysis using Synthetic Datasets from RD-Connect GPAP)' [19] to teach users how to access data from the EGA and to recreate and run the workflow from scratch. In addition, the workflow is available at WorkflowHub [20].

1 Possible via https://ega.ebi.ac.uk:8443/ega-openid-connect-server/ega-login

2 https://github.com/galaxyproject/galaxy/issues/14578

Table 1. Overview of existing Galaxy trio analysis tools and the number of variants they report.

Family	GEMINI	gene.iobio
Case1	0	1
Case2	77	1
Case3	0	2
Case4	26	1
Case5	142	1
Case6	0	1

Use Case: Breast Cancer

Here we report on the output produced by gene.iobio to demonstrate it's added value to the Galaxy platform. To produce these results we used case 5 from the EGA dataset. This case describes a family trio where the mother and daughter are affected by breast cancer. The case describes an autosomal dominant inheritance pattern, which causes a missense single nucleotide polymorphism (SNP) at chromosome 17 position 41,215,920 changing a guanine into a thymine. [17]

The BAMs and VCFs of the family trio are first downloaded using the PyEGA3 tool in Galaxy. The tool was able to securely download the trios' VCFs and slices of the large BAMs by selecting chromosome 17. After downloading the data from the EGA the workflow preprocesses the data and produces multiple outputs using gene.iobio.

Firstly, a disease/phenotype of interest can be provided to produce a list of genes of interest. To generate this list of genes the gene.iobio makes use of the Phenolyzer software [21]. In this case the disease is breast cancer. The automatic selection of important genes related to the disease speeds up the process of finding causative variants. Alternatively, genes can be added manually.

Next, gene.iobio searches, by default, for causative variants in the top 20 of provided list of genes by filtering all the variants in the VCFs using pre-selected, but customizable, parameters. Figure 2 shows that a spiked-in causative variant was found with sufficient depth and allele counts. In addition, gene.iobio shows the quality of the variant, a pathogenicity score, the population frequency, a visualization of the inhertiance patterns, and statistics on the conservation of the variant. This information helps the user to determine the legitimacy of the variant.

Overall, gene.iobio provides an interactive and visual overview of causative variant identification. This is a significant improvement compared to the previous causative variant identification tool GEMINI. Especially with regards to identifying the quality of the causative variant as illustrated by figure 2.

Trio Analysis Comparison

In addition, we further validated the gene.iobio tool by identifying the causative variants in all the families available. A comparison of the results reported by gene.iobio and GEMINI is shown in table 1. It shows the number of variants reported by GEMINI and gene.iobio using the default parameters. The table shows that GEMINI does not report any variants for some cases. When GEMINI does report variants it reports the correct variants. However, it also reports many false positive, since each family has only one or two spiked-in causative variants. In contrast, gene.iobio does report causative variants for each family and only the correct ones. This shows that gene.iobio is not only interpretable but also accurate.

Limitations & Future Work

In the current implementation of PyEGA3 in Galaxy we miss the support of authentication with Passports, a Global Alliance for Genomics and Health (GA4GH) standard. The GA4GH has developed a set of standards to facilitate data sharing within a federated context. To access federated resources and controlled access data, the identity of the user accessing the data must be determined, along with any data access permissions the user has for particular datasets. Two GA4GH standards facilitate this, the AAI standard, and the Passport standard. The AAI specification profiles OpenID Connect (OIDC) protocol to provide a mechanism for interoperability of identities between different institutions, supporting federated data access while ensuring the security of the data by defining the way identities and access permissions are exchanged between resources. The Passport standard defines how the permissions are represented, in the form of visas. There are 5 types of visa, ControlledAccessGrants which list the access permissions for the user to controlled access datasets, LinkedIdentities which allow a user to link different identities to facilitate single sign on, as well as AffiliationAndRole, AcceptedTermsAndPolicies, and ResearcherStatus. Passports support tiered access - open, registered, and controlled. Typically, the data available to the user will increase and the user moves from open to controlled access. Any user can access resources on the open access tier, while ResearcherSatus indicates the user can access resources at the registered access tier, and ControlledAccessGrants indicate which controlled access resources the user can access. The Life Science AAI supports GA4GH AAI and Passport standards. This means that a user can link their Life Science identity with one or more institutional or social media identities, and utilise these identities to access resources, such as Galaxy instances or datasets from the EGA. For example, a user can use their linked institutional identity via the Life Science AAI to access data from EGA via the EGA Permissions API and Data API. In the future, we aim to implement the Passport protocol into Galaxy to access data compliant with the GA4GH standards [22].

Conclusion

In this study we implemented PyEGA3 in Galaxy to retrieve data from the EGA in a GA4GH compliant manner. In addition, gene.iobio was implemented to improve variant analyses in Galaxy. These tools were validated by using B1MG data from the EGA and creating a findable analysis workflow into Galaxy. This work illustrates that gene.iobio is a major improvement compared to the current trio analysis tool in Galaxy as it creates interpretable and dynamic plots. In addition, we showed that Galaxy makes it feasible and manageable for any researcher to retrieve data from the EGA securely and analyse family trio data in a FAIR manner. Not only is this work applicable to trio analysis, it is also transferable to other omics analysis, such as genome assembly, metabolomics, metagenomics, proteomics, and transcriptomics. In conclusion, this work illustrates that Galaxy is one step closer to becoming a generalised omics platform for FAIR data analysis.

Methods

Implementation

The installation and dependencies for gene.iobio are handled by Galaxy. The version of gene.iobio reported here is v4.7.1a.

Training Materials

Our workflow greatly simplifies the data collection from the EGA and the visualization and analysis of family trios. In addition, we created a tutorial for running the workflow on Galaxy. Also, the tutorial describes in detail how to gain access to datasets on the EGA to simplify the adoption of this workflow for other data on the EGA. The tutorial is available at the Galaxy training materials website [19].

figures/figure2.pdf

Figure 2. Overview of gene.iobio results for the spiked-in variant. The figure shows statistics on quality of the variant, phenotype associations, pathogenicity, population frequency, inheritance, and conservation.

Pre-Processing

Firstly, a ‘chr‘ prefix is added to the first column of each chromosomal site in the VCFs, to match it with the built-in reference genome from Galaxy (hg19). Secondly, the VCFs are normalized using bcftools [23]. The normalization process includes left aligning insertion or deletion and splitting multiallelic sites into biallelic records. Thirdly, the VCFs in the EGA dataset are actually genomic variant calling format (GVCFs) files. A GVCF has a record for (almost) all sites even when no variant, denoted by <NON_REF>, is recorded. In this study this information is not informative and slows down analysis. Therefore, the records with a <NON_REF> site are filtered out. Fourthly, the VCFs are merged into a single VCF based on their trio pairing using bcftools. This creates a VCF where each record also has a presence/absence column for each family pair. Finally, the variants in the merged VCF are filtered and annotated using the SnpEff tool [4] as required by GEMINI.

gene.iobio

Gene.iobio is run with the GRCh37 reference genome. The gene list is created using the phenotypes described in Additional file 1. The default search filters are used to detect the causative variant.

GEMINI

For each case GEMINI is prompted to remove low impact severity variants and to search for causative variants that match the inheritance pattern in Additional file 1.

Availability of source code and requirements

PyEGA3

- Project name: pyega3
- Github repository: https://github.com/galaxyproject/tools-iuc/tree/master/tools/pyega3
- ToolShed repository: https://toolshed.g2.bx.psu.edu/view/iuc/ega_download_client
- Training Manual: https://training.galaxyproject.org/training-material/topics/variant-analysis/tutorials/trio-analysis/tutorial.html
- Operating system(s): Unix (Platform independent with Docker, Singularity)
- Other requirements: Galaxy version 22.05 or higher
- License: MIT

gene.iobio

- Project name: gene.iobio
- Github repository: https://github.com/galaxyproject/tools-iuc/tree/master/tools/geneiobio
- ToolShed repository: https://toolshed.g2.bx.psu.edu/view/iuc/geneiobio
- Training Manual: https://training.galaxyproject.org/training-material/topics/variant-analysis/tutorials/trio-analysis/tutorial.html
- Operating system(s): Unix (Platform independent with Docker, Singularity)
- Other requirements: Galaxy version 22.05 or higher
- License: MIT

Galaxy Resources

- Galaxy Home Page: https://galaxyproject.org/
- Galaxy Tutorials: https://training.galaxyproject.org
- How to install Galaxy: https://getgalaxy.org
- How to install tools: https://galaxyproject.org/admin/tools/add-tool-from-toolshed-tutorial/
- Full administrative resources: https://docs.galaxyproject.org/
- Galaxy Help Forum: https://help.galaxyproject.org/
- Connect with the Galaxy Community on Gitter Chat: https://gitter.im/galaxyproject/Lobby/

Availability of supporting data and materials

The data used in this study were generated by a public human WGS experiment in the Illumina Platinum initiative [24], which was made available by the HapMap project [25]. All data from this project is available at the EGA website [12]. In this study only the BAMs and VCFs with the chromosomes containing the spiked-in variants were included. These files are available under 'RD-Connect GPAP synthetic data spiked-in variant data' at Zenodo [26].

Additional Files

Additional file 1. The report describing the family trios.

Declarations

List of abbreviations

AAI: Authentication and Authorisation; BAM: Binary alignment map; B1MG: Beyond 1 Million Genomes; DAC: Data Access Committee; EGA: European Genome-Phenome Archive; FAIR: Findable, Accessible, Interoperable, and Reusable; GA4GH: Global Alliance for Genomics and Health; GTN: Galaxy Training Network; GVCF: Genomic variant calling format; OIDC: OpenID Connect; SNP: Single nucleotide polymorphism; VCF: Variant call format.

Competing Interests

The authors declare that they have no competing interests.

Funding

This work This project has received funding from the EC H2020 project CINECA (grant 825775) [27] as well as the Erasmus+ programme of the European Union (Gallantries Project, grant 2020-1-NL01-KA203-064717, doi:10.13039/100001501). The data used in this project was created with the support of the RD-Connect GPAP [28], EC H2020 project EJP-RD (grant 825575) [29], EC H2020 project B1MG (grant 951724) [30], and Generalitat de Catalunya project VEIS (grant 001-P-001647) [31].

Author's Contributions

J.O., S.H., H.R., and D.S. contributed to writing the manuscript. J.O., S.H., H.R. contributed to writing the Galaxy workflow tutorial. S.H., H.R., and D.D. contributed to implementing the PyEGA3 and gene.iobio tool in Galaxy. A.P.S. supervised the project. All authors approved the manuscript.

Acknowledgements

We would like to thank the Galaxy community and in particular Wolfgang Maier, for reviewing, testing, and validating the workflow tutorial, and PyEGA3.

References

1. Inau ET, Sack J, Waltemath D, Zeleke AA. Initiatives, Concepts, and Implementation Practices of FAIR (Findable, Accessible, Interoperable, and Reusable) Data Principles in Health Data Stewardship Practice: Protocol for a Scoping Review. JMIR Research Protocols 2021;10:e22505.
2. Wilkinson MD, Dumontier M, Aalbersberg IJ, Appleton G, Axton M, Baak A, et al. The FAIR Guiding Principles for scientific data management and stewardship. Scientific Data 2016;3.
3. Nicorici D, Şatalan M, Edgren H, Kangaspeska S, Murumagi A, Kallioniemi O, et al. FusionCatcher: a tool for finding somatic fusion genes in paired-end RNA-sequencing data. bioRxiv 2014;.
4. Cingolani P, Platts A, Wang LL, Coon M, Nguyen T, Wang L, et al. A program for annotating and predicting the effects of single nucleotide polymorphisms, SnpEff. Fly 2012;6:80–92.
5. Danecek P, Bonfield JK, Liddle J, Marshall J, Ohan V, Pollard MO, et al. Twelve years of SAMtools and BCFtools. GigaScience 2021;10.
6. Krzywinski M, Schein J, Birol İ, Connors J, Gascoyne R, Horsman D, et al. Circos: An information aesthetic for comparative genomics. Genome Research 2009;19:1639–1645.
7. Jalili V, Afgan E, Gu Q, Clements D, Blankenberg D, Goecks J, et al. The Galaxy platform for accessible, reproducible and collaborative biomedical analyses: 2020 update. Nucleic Acids Research 2020;48:W395–W402.
8. Goble C, Soiland-Reyes S, Bacall F, Owen S, Williams A, Eguinoa I, et al., Implementing FAIR Digital Objects in the EOSC-Life Workflow Collaboratory.; 2021. https://zenodo.org/record/4605654, accessed: 2022-03-21.

9. Galaxy Training Network Stats;. https://training.galaxyproject.org/stats, accessed: 2022-10-27.
10. Rasche H, Hiltemann S. Galactic Circos: User-friendly Circos plots within the Galaxy platform. GigaScience 2020;9.
11. Lappalainen I, Almeida-King J, Kumanduri V, Senf A, Spalding JD, ur Rehman S, et al. The European Genome-phenome Archive of human data consented for biomedical research. Nature Genetics 2015;47(7):692–695.
12. Freeberg MA, Fromont LA, D'Altri T, Romero AF, Ciges J, Jene A, et al. The European Genome-phenome Archive in 2021. Nucleic Acids Research 2021;50:D980–D987.
13. Beyond 1 Million Genomes;. https://b1mg-project.eu/, accessed: 2022-06-30.
14. Sera TD, Velinder M, Ward A, Qiao Y, Georges S, Miller C, et al. Gene.iobio: an interactive web tool for versatile, clinically-driven variant interrogation and prioritization. Scientific Reports 2021;11.
15. Hiltemann S, Rasche H, Gladman S, Hotz HR, Larivière D, Blankenberg D, et al. Galaxy Training: A powerful framework for teaching! PLOS Computational Biology 2023;19(1):1–18.
16. Paila U, Chapman BA, Kirchner R, Quinlan AR. GEMINI: Integrative Exploration of Genetic Variation and Genome Annotations. PLOS Computational Biology 2013;9:1–8.
17. Rare Disease Synthetic Dataset;. https://ega-archive.org/datasets/EGAD00001008392, accessed: 2022-10-06.
18. Galaxy Training Network Stats;. https://training.galaxyproject.org/, accessed: 2022-10-27.
19. Trio Analysis using Synthetic Datasets from RD-Connect GPAP;. https://training.galaxyproject.org/training-material/topics/variant-analysis/tutorials/trio-analysis/tutorial.html, accessed: 2022-09-23.
20. Trio Analysis;. https://workflowhub.eu/workflows/363, accessed: 2023-03-21.
21. Yang H, Robinson PN, Wang K. Phenolyzer: phenotype-based prioritization of candidate genes for human diseases. Nature Methods 2015;12:841–843.
22. Voisin C, Linden M, Dyke SOM, Bowers SR, Alper P, Barkley MP, et al. GA4GH Passport standard for digital identity and access permissions. Cell Genomics 2021;1(2):100030.
23. Danecek P, Bonfield JK, Liddle J, Marshall J, Ohan V, Pollard MO, et al. Twelve years of SAMtools and BCFtools. GigaScience 2021;10.
24. Eberle MA, Fritzilas E, Krusche P, Källberg M, Moore BL, Bekritsky MA, et al. A reference data set of 5.4 million phased human variants validated by genetic inheritance from sequencing a three-generation 17-member pedigree. Genome Research 2016;27:157–164.
25. HapMap Project;. www.genome.gov/10001688/international-hapmap-project, accessed: 2022-11-02.
26. Ouwerkerk J, Zenodo: RD-Connect GPAP synthetic data; 2022. https://doi.org/10.5281/zenodo.7274482.
27. CINECA Project;. https://cordis.europa.eu/project/id/825775, accessed: 2022-11-02.
28. RD-Connect GPAP Project;. https://platform.rd-connect.eu/, accessed: 2022-11-02.
29. EJP-RD Project;. https://cordis.europa.eu/project/id/825575, accessed: 2022-11-02.
30. B1MG Project;. https://cordis.europa.eu/project/id/951724, accessed: 2022-11-02.
31. VEIS Project;. http://www.gcatbiobank.org/investigadors/projects/25/veis-european-project-valuation-of-the-european-archive-of-the-genome-phenome-ega-for-the-industry-and-the-society, accessed: 2022-11-02.

Click here to access/download;Figure;figure2.pdf

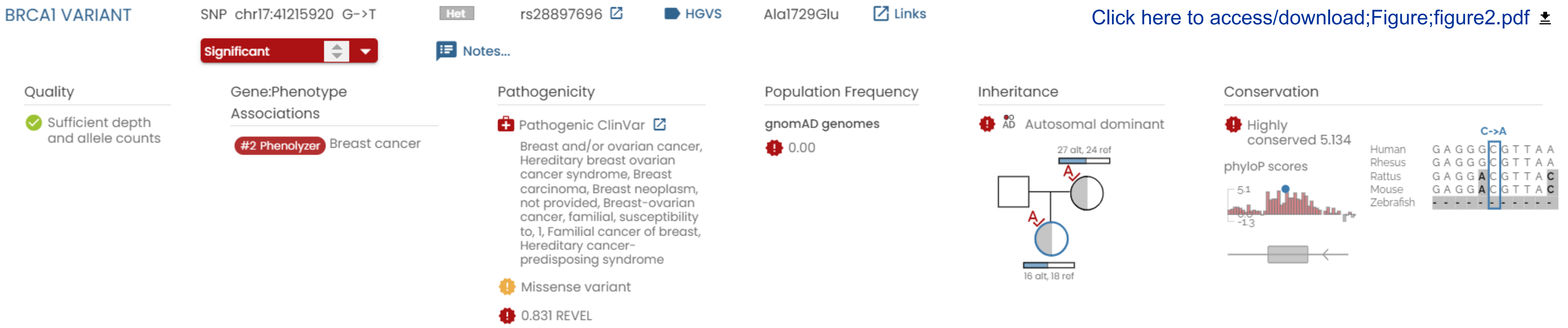

Click here to access/download;Figure;figure1.pdf

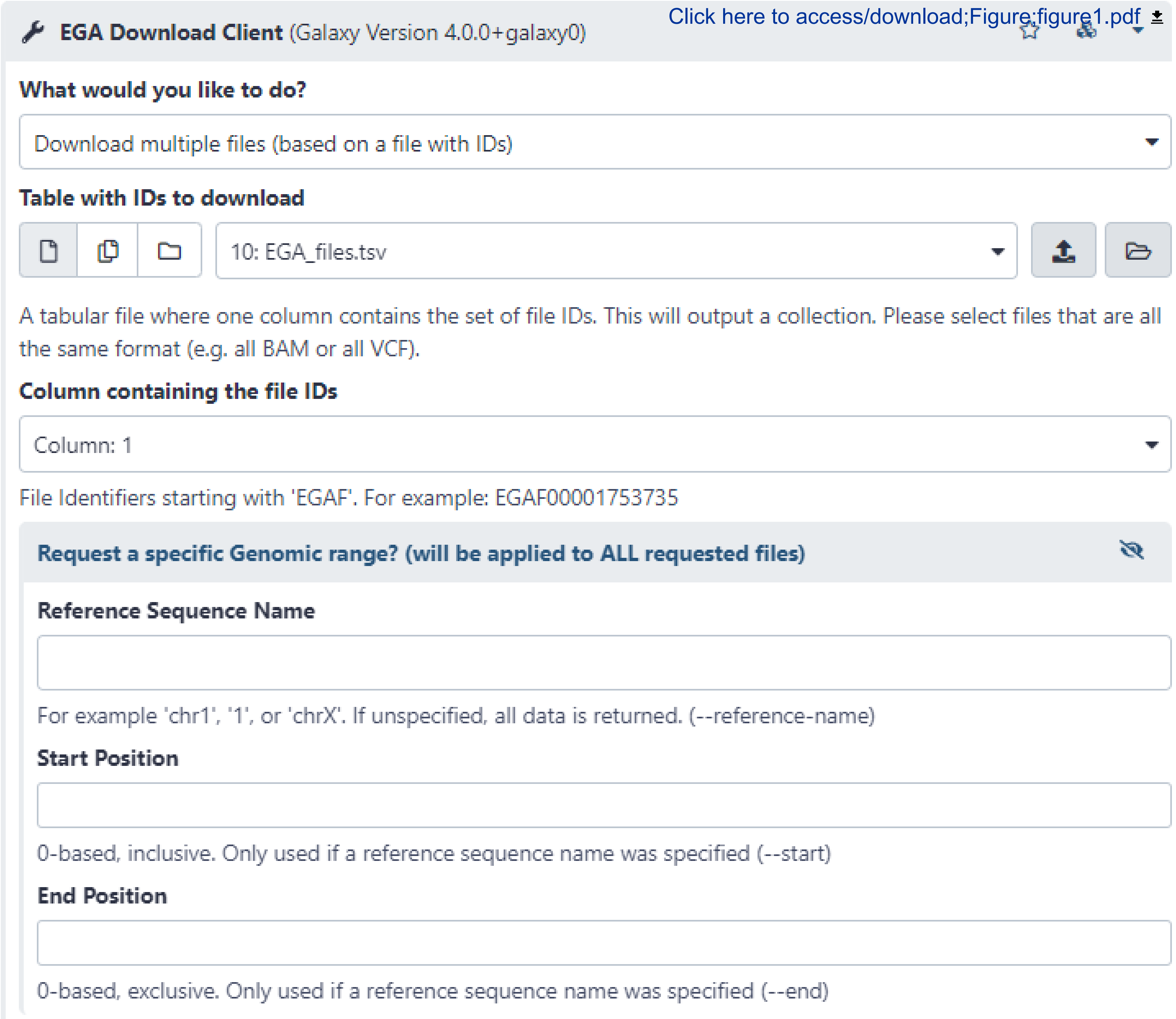

Click here to access/download
Supplementary Material
Additional file 1.pdf
