# Supplementary material for: FAIR data retrieval for sensitive clinical research data in Galaxy
Source: Gigascience. 2024 Jan 27;13:giad099. doi: 10.1093/gigascience/giad099 (PMC10821763; doi:10.1093/gigascience/giad099)
Supplement: giad099_GIGA-D-23-00177_Revision_2 [file giad099_giga-d-23-00177_revision_2.pdf]

GigaScience
FAIR Data Retrieval for Sensitive Clinical Research Data in Galaxy
--Manuscript Draft--

**Manuscript Number:**	GIGA-D-23-00177R2
**Full Title:**	FAIR Data Retrieval for Sensitive Clinical Research Data in Galaxy
**Article Type:**	Technical Note
**Funding Information:**	Horizon 2020 (825775) — Dr Andrew Peter Stubbs<br>Erasmus+ (2020-1-NL01-KA203-064717) — Dr Andrew Peter Stubbs
**Abstract:**	Background, In clinical research, data has to be accessible and reproducible, but the generated data is becoming larger and analysis complex. Here we propose a platform for FAIR data access and creating reproducible findings. Standardised access to a major genomic repository, the European Genome-Phenome Archive (EGA), has been achieved with API services like PyEGA3. We aim to provide a FAIR data analysis service in Galaxy by retrieving genomic data from the EGA and provide a generalised "omics" platform for FAIR data analysis.<br>Results, To demonstrate this, we implemented an end-to-end Galaxy workflow to replicate the findings from an RD-Connect synthetic dataset Beyond the 1 Million Genomes (synB1MG) available from the EGA. We developed the PyEGA3 connector within Galaxy to easily download multiple datasets from the EGA. We added the gene.iobio tool, a diagnostic environment for precision genomics, to Galaxy and demonstrate that it provides a more dynamic and interpretable view for trio analysis results. We developed a Galaxy trio analysis workflow to determine the pathogenic variants from the synB1MG trios using the GEMINI and gene.iobio tool. The complete workflow is available at WorkflowHub and an associated tutorial was created in the Galaxy Training Network which helps researchers unfamiliar with Galaxy to run the workflow.<br>Conclusion, We showed the feasibility of reusing data from the EGA in Galaxy via PyEGA3 and validated the workflow by re-discovering spiked-in variants in synthetic data. Finally, we improved existing tools in Galaxy and created a workflow for trio analysis to demonstrate the value of FAIR genomics analysis in Galaxy.
**Corresponding Author:**	Jasper Ouwerkerk<br>Erasmus MC<br>Rotterdam, NETHERLANDS
**Corresponding Author Secondary Information:**	
**Corresponding Author's Institution:**	Erasmus MC
**Corresponding Author's Secondary Institution:**	
**First Author:**	Jasper Ouwerkerk
**First Author Secondary Information:**	
**Order of Authors:**	Jasper Ouwerkerk<br>Helena Rasche<br>John Dylan Spalding<br>Saskia Hiltemann<br>Andrew Peter Stubbs
**Order of Authors Secondary Information:**	
**Response to Reviewers:**	Dear editors,<br><br>Thank you for reviewing and provisionally accepting our manuscript. We have updated

Powered by Editorial Manager® and ProduXion Manager® from Aries Systems Corporation

	the manuscript to address the suggested edits. We hope the manuscript is now ready for publication in the GigaScience journal.<br><br>Kind regards,<br><br>Jasper Ouwerkerk (MSc)<br>PhD candidate<br>Department of Pathology & Clinical Bioinformatics<br><br>On behalf of all authors.<br><br><br>Editor:<br><br>Your manuscript "FAIR Data Retrieval for Sensitive Clinical Research Data in Galaxy" (GIGA-D-23-00177R1) has been assessed by our reviewers. Based on these reports, and my own assessment as Editor, I am pleased to inform you that it is potentially acceptable for publication in GigaScience, once you have carried out some essential formatting revisions suggested by our Editor.<br>Please see the attached marked up PDF with comments. Note the GigaDB DOI citation needs to be added to the paper and also the References - this has been marked up.<br>We have updated our manuscript according to your comments. We added the RRIDs where needed and the GigaDB citation of our supporting data to the manuscript.
**Additional Information:**	
**Question**	**Response**
Are you submitting this manuscript to a special series or article collection?	No
**Experimental design and statistics**<br><br><br>Full details of the experimental design and statistical methods used should be given in the Methods section, as detailed in our Minimum Standards Reporting Checklist. Information essential to interpreting the data presented should be made available in the figure legends.<br><br><br>Have you included all the information requested in your manuscript?	Yes
**Resources**<br><br><br>A description of all resources used, including antibodies, cell lines, animals and software tools, with enough information to allow them to be uniquely identified, should be included in the Methods section. Authors are strongly encouraged to cite Research Resource Identifiers (RRIDs) for antibodies, model organisms and tools, where possible.	Yes

Have you included the information requested as detailed in our Minimum Standards Reporting Checklist?	
**Availability of data and materials**<br><br>All datasets and code on which the conclusions of the paper rely must be either included in your submission or deposited in publicly available repositories (where available and ethically appropriate), referencing such data using a unique identifier in the references and in the “Availability of Data and Materials” section of your manuscript.<br><br>Have you have met the above requirement as detailed in our Minimum Standards Reporting Checklist?	Yes

Click here to access/download;LaTeX - Main Document (TeX file);main.tex

```
This is pdfTeX, Version 3.141592653-2.6-1.40.24 (TeX Live 2022)
(preloaded format=pdflatex 2023.3.8)  27 OCT 2023 03:46
entering extended mode
 restricted \write18 enabled.
 %&-line parsing enabled.
**main.tex
(./main.tex
LaTeX2e <2022-11-01> patch level 1
L3 programming layer <2023-02-22>

! LaTeX Error: File `oup-contemporary.cls' not found.

Type X to quit or <RETURN> to proceed,
or enter new name. (Default extension: cls)

Enter file name:
! Emergency stop.
<read *>

l.11 ^^M

*** (cannot \read from terminal in nonstop modes)


Here is how much of TeX's memory you used:
 23 strings out of 476024
 503 string characters out of 5794017
 1849382 words of memory out of 5000000
 20571 multiletter control sequences out of 15000+600000
 512287 words of font info for 32 fonts, out of 8000000 for 9000
 1141 hyphenation exceptions out of 8191
 19i,0n,29p,95b,17s stack positions out of
10000i,1000n,20000p,200000b,200000s
!  ==> Fatal error occurred, no output PDF file produced!
```

Manuscript
Click here to access/download;Manuscript;main.pdf

GigaScience, 2017, 1–6

doi: xx.xxxx/xxxx
Manuscript in Preparation
Paper

PAPER

FAIR Data Retrieval for Sensitive Clinical Research Data in Galaxy

Jasper Ouwerkerk[1,*,†], Helena Rasche[1,†], John D. Spalding[2], Saskia Hiltemann[1,††] and Andrew P. Stubbs[1,††]

[1]Erasmus Medical Center, Clinical Bioinformatics Group, Department of Pathology, Wytemaweg 80, 3015 CN, Rotterdam, The Netherlands and [2]CSC–IT Center for Science, Keilaranta 14, 02101, Espoo, Finland

[*]Correspondence address. Jasper Ouwerkerk, Clinical Bioinformatics Group, Department of Pathology, Erasmus Medical Center, Wytemaweg 80, 3015 CN, Rotterdam, The Netherlands. E-mail: j.ouwerkerk.1@erasmusmc.nl
[†]Contributed equally.

Abstract

Background, In clinical research, data has to be accessible and reproducible, but the generated data is becoming larger and analysis complex. Here we propose a platform for FAIR data access and creating reproducible findings. Standardised access to a major genomic repository, the European Genome-Phenome Archive (EGA), has been achieved with API services like PyEGA3. We aim to provide a FAIR data analysis service in Galaxy by retrieving genomic data from the EGA and provide a generalised "omics" platform for FAIR data analysis.
Results, To demonstrate this, we implemented an end-to-end Galaxy workflow to replicate the findings from an RD-Connect synthetic dataset Beyond the 1 Million Genomes (synB1MG) available from the EGA. We developed the PyEGA3 connector within Galaxy to easily download multiple datasets from the EGA. We added the gene.iobio tool, a diagnostic environment for precision genomics, to Galaxy and demonstrate that it provides a more dynamic and interpretable view for trio analysis results. We developed a Galaxy trio analysis workflow to determine the pathogenic variants from the synB1MG trios using the GEMINI and gene.iobio tool. The complete workflow is available at WorkflowHub and an associated tutorial was created in the Galaxy Training Network which helps researchers unfamiliar with Galaxy to run the workflow.
Conclusions, We showed the feasibility of reusing data from the EGA in Galaxy via PyEGA3 and validated the workflow by re-discovering spiked-in variants in synthetic data. Finally, we improved existing tools in Galaxy and created a workflow for trio analysis to demonstrate the value of FAIR genomics analysis in Galaxy.

Key words: B1MG; FAIR; Galaxy; Trio Analysis

Findings

Background

In the last few years there have been many developments in Findable, Accessible, Interoperable, and Reusable (FAIR) data [1]. FAIR data is data and corresponding metadata which are 1) findable by both machines and humans, 2) accessible using a standard open protocol, 3) interoperable so it can easily be processed and analysed, 4) resuable so the data can be understood by anyone and make analyses reproducible [2]. FAIR data allows researchers to reanalyse data with new genetic analysis tools not yet available at the time of data publication. For example, in a study on fusion genes, 24 novel fusion in breast cancer were found with the introduction of a new tool [3].

However, for many biomedical analyses, researchers are required to have considerable knowledge on using analysis tools. Moreover, many tools require knowledge of Unix commands or python coding. [4, 5, 6]. This creates a barrier for clinical researchers that want to reanalyse data, reducing the adoption of implementing FAIR principles and reanalyses.

Compiled on: October 27, 2023.
Draft manuscript prepared by the author.

Key Points

- Secure access to GA4GH EGA service using PyEGA3 Galaxy service
- Standard analysis for B1MG synthetic data
- Interactive gene variant detection for trio analysis with gene.iobio in Galaxy
- Created a tutorial associated with the Galaxy Training Network

The Galaxy platform [7] supports researchers in adopting these complex computation tools for their FAIR data analysis. Galaxy is an online analysis platform with a plethora of tools to perform text/table processing, omics analysis, machine learning, image analysis, and more. All these tools are maintained and developed by a growing community. Using the tools does not require any programming skills and are easy to share with colleagues and other researchers. At the end of the analysis the workflow of tools can be exported to reproduce the analysis [7]. These workflows can be made discoverable by uploading the workflow to WorkflowHub [8], a registry for describing, sharing, and publishing scientific computational workflows. In addition, Galaxy already has 300+ tutorials describing workflows on genome assembly, ecology, metagenomics, variant analysis and more [9]. This is beneficial to many researchers since there are many complex Unix-based tools which are essential for biomedical research. An example of such an application is Circos [6], which is a complicated visualization tool for comparing whole genomes. This tool has been implemented within Galaxy, which makes it simple for any researcher to create Circos plots [10].

Even though Galaxy is a well-established platform for analysis, it still lacks applications for retrieving access-controlled data from large repositories like the EGA. The EGA controls the accessibility to datasets using Data Access Committees (DACs). Requestors can access data from the EGA by contacting the DAC for the dataset of interest. DACs are generally formed by the organization which collected the data and performed the analysis. This allows researchers to access datasets of interest and also manage the accessibility of their data at the EGA [11].

In this work we implemented PyEGA3 [12], a tool which can access controlled data from the EGA, within Galaxy. Here access to datasets is managed via the EGA. Our implementation of the PyEGA3 tool allows to filter datasets, available on the EGA, based on their metadata and scale up analysis. This will be showcased by validating our workflow for trio analysis on family trios from the Beyond 1 Million Genomes (B1MG) project [13]. In trio analysis the differences in DNA between the maternal, paternal, and affected child i.e., proband, is analysed to detect causative variants causing a particular disease in the proband. To perform the trio-analysis we added gene.iobio, a standalone web-based tool, to Galaxy [14]. The complete workflow, including data retrieval with PyEGA3, is implemented within Galaxy and uploaded to WorkflowHub for discoverability. In addition, we wrote a tutorial to explain our workflow in detail, which is associated with the Galaxy Training Network (GTN) [15]. This study shows it is feasible to adopt end-to-end scalable FAIR analysis of clinical data, and ultimately for any future analysis on data available at the EGA.

Results

PyEGA3

PyEGA3 was implemented to retrieve access controlled data from the EGA in Galaxy. Authentication of the user is done by password and username. This information is encrypted using a Vault abstraction [16] when configured by the Galaxy administrator. In addition, LS Login (Previously ELIXIR Authentication and Authorization Infrastructure (AAI)) tokens can be used for authentication, if setup by the user. The tokens are stored in the Galaxy database and temporarily valid (one hour by default) to shorten the window of time for a potential attack. Currently, the process of authentication is initiated by linking one's EGA account to their LS Login[1] (previously ELIXIR AAI) account. Next, the user logs in to Galaxy via LS Login, which attaches the user's GA4GH passport and access and refresh tokens to the user's account in Galaxy. The refresh token is used to regularly refresh their credentials allowing the Galaxy server to act on their behalf when the user requests it via tool execution. Upon executing a tool, assuming the tool is written to support it, the access token, or possibly in the future passports, are attached to the tool's execution such that they can be used to authenticate the user. While currently the access token is implemented on an ad-hoc basis, we intend to directly implement support for this type of tool and authentication method in a future version of Galaxy. [2]

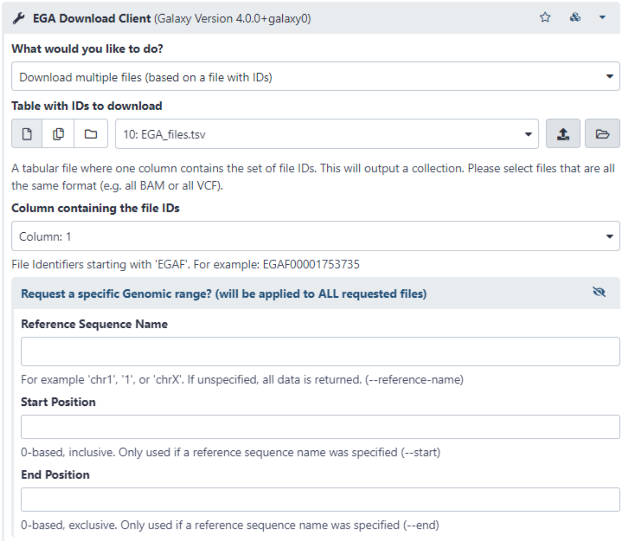

Figure 1. The Galaxy interface of the added feature to the PyEga3 tool to download multiple files. It takes a tabular data with EGAF IDs. In addition, a region can be provided to download a small region in BAMs or VCFs.

The tool implemented in Galaxy has the same functionalities as the command line version, namely list a user's authorized datasets, list files in a dataset and fetch a file or all files in a dataset. In addition, we added the option to download a specified list of files from the EGA. With this option it is still possible to download a specific genomic range, see Figure 1, which is useful for large binary alignment map (BAM) and variant calling format (VCF) files.

Gene.iobio

We also implemented the gene.iobio tool within the Galaxy framework. Gene.iobio is a tool for precision genomics. The tool is able to create dynamic results, which include creating a list of genes for

1 Possible via https://ega.ebi.ac.uk:8443/ega-openid-connect-server/ega-login
2 https://github.com/galaxyproject/galaxy/issues/14578

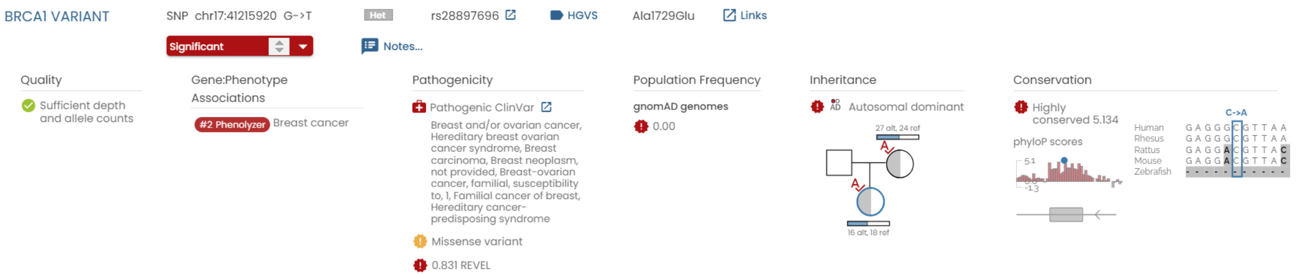

Figure 2. Overview of gene.iobio results for the spiked-in variant. The figure shows statistics on quality of the variant, phenotype associations, pathogenicity, population frequency, inheritance, and conservation.

the disease of interest, creating an automatic report of pathogenic variants within the list of genes, allowing the custom filtering of pathogenic variants, reporting phenotypes and publications related to the gene of interest, and reviewing the variants. These are major improvements compared to the existing trio analysis tool within Galaxy, GEMINI [17], which was only able to produce static plots or large lists of filtered variants.

Workflow & Tutorial

In this study we illustrate an end-to-end workflow for trio analysis for FAIR data. This workflow retrieves and analyses files from large datasets in the EGA and can easily be adapted to any other EGA dataset. We illustrate this by analysing data from the EGAD00001008392 [18] dataset. This dataset contains 6 trio families with different inheritance patterns of digitally spiked-in variants, where each family is subject to a different disease. Next, we demonstrate the usefulness of gene.iobio by analysing the family trios and comparing the existing trio analysis tool in Galaxy, GEMINI, to the gene.iobio tool. Finally, a comprehensive tutorial is made available at the Galaxy Training materials [19] under the topic 'Variant Analysis' titled 'Trio Analysis using Synthetic Datasets from RD-Connect GPAP)' [20] to teach users how to access data from the EGA and to recreate and run the workflow from scratch. In addition, the workflow is available at WorkflowHub [21].

Use Case: Breast Cancer

Here we report on the output produced by gene.iobio to demonstrate it's added value to the Galaxy platform. To produce these results we used case 5 from the EGA dataset. This case describes a family trio where the mother and daughter are affected by breast cancer. The case describes an autosomal dominant inheritance pattern, which causes a missense single nucleotide polymorphism (SNP) at chromosome 17 position 41,215,920 changing a guanine into a thymine. [18]

The BAMs and VCFs of the family trio are first downloaded using the PyEGA3 tool in Galaxy. The tool was able to securely download the trios' VCFs and slices of the large BAMs by selecting chromosome 17. After downloading the data from the EGA the workflow preprocesses the data and produces multiple outputs using gene.iobio.

Firstly, a disease/phenotype of interest can be provided to produce a list of genes of interest. To generate this list of genes the gene.iobio makes use of the Phenolyzer software [22]. In this case the disease is breast cancer. The automatic selection of important genes related to the disease speeds up the process of finding causative variants. Alternatively, genes can be added manually.

Next, gene.iobio searches, by default, for causative variants in the top 20 of provided list of genes by filtering all the variants in the VCFs using pre-selected, but customizable, parameters. Figure 2 shows that a spiked-in causative variant was found with sufficient depth and allele counts. In addition, gene.iobio shows the quality of the variant, a pathogenicity score, the population frequency, a visualization of the inhertiance patterns, and statistics on the conservation of the variant. This information helps the user to determine the legitimacy of the variant.

Table 1. Overview of existing Galaxy trio analysis tools and the number of variants they report.

Family	GEMINI	gene.iobio
Case1	0	1
Case2	77	1
Case3	0	2
Case4	26	1
Case5	142	1
Case6	0	1

Overall, gene.iobio provides an interactive and visual overview of causative variant identification. This is a significant improvement compared to the previous causative variant identification tool GEMINI. Especially with regards to identifying the quality of the causative variant as illustrated by figure 2.

Trio Analysis Comparison

In addition, we further validated the gene.iobio tool by identifying the causative variants in all the families available. A comparison of the results reported by gene.iobio and GEMINI is shown in table 1. It shows the number of variants reported by GEMINI and gene.iobio using the default parameters. The table shows that GEMINI does not report any variants for some cases. When GEMINI does report variants it reports the correct variants. However, it also reports many false positive, since each family has only one or two spiked-in causative variants. In contrast, gene.iobio does report causative variants for each family and only the correct ones. This shows that gene.iobio is not only interpretable but also accurate.

Limitations & Future Work

PyEGA Passports

In the current implementation of PyEGA3 in Galaxy we miss the support of authentication with Passports, a Global Alliance for Genomics and Health (GA4GH) standard. The GA4GH has developed a set of standards to facilitate data sharing within a federated context. To access federated resources and controlled access data, the identity of the user accessing the data must be determined, along with any data access permissions the user has for particular datasets. Two GA4GH standards facilitate this, the AAI standard, and the Passport standard. The AAI specification profiles OpenID Connect (OIDC) protocol to provide a mechanism for interoperability of identities between different institutions, supporting federated data access while ensuring the security of the data by defining the way identities and access permissions are exchanged between resources. The Passport standard defines how the permissions are represented, in the form of visas. There are 5 types of visa, ControlledAccessGrants which list the access permissions for the user to controlled access datasets, LinkedIdentities which allow a user to link different identities to facilitate single sign on, as well as AffiliationAnd-

Role, AcceptedTermsAndPolicies, and ResearcherStatus. Passports support tiered access - open, registered, and controlled. Typically, the data available to the user will increase and the user moves from open to controlled access. Any user can access resources on the open access tier, while ResearcherSatus indicates the user can access resources at the registered access tier, and ControlledAccessGrants indicate which controlled access resources the user can access. The Life Science AAI supports GA4GH AAI and Passport standards. A user can link their Life Science identity with one or more institutional or social media identities, and utilise these identities to access resources, such as Galaxy instances or datasets from the EGA. For example, a user can use their linked institutional identity via the Life Science AAI to access data from EGA via the EGA Permissions API and Data API. In the future, we aim to implement the Passport protocol into Galaxy to access data compliant with the GA4GH standards [23].

Data Management

In addition to secure data retrieval, Galaxy is working on improving secure data management. Currently, data is stored unencrypted on the user's Galaxy account. But, with PyEGA implemented in Galaxy, confidential data from the EGA could be left unprotected when uploaded to a public Galaxy server. This would violate the EGA Data Access Agreement (DAA) which requires the user's institution to preserve the confidentiality of the data.

Our approach for processing confidential human genetic data would currently only be in compliance with EGA guidelines and the General Data Protection Regulation (GDPR) [24] when data is stored on a private Galaxy managed by the user's institution. However, data privacy at rest does exist within the community, currently S3 buckets [25] can be leveraged by Galaxy, which offer the ability to encrypt data at rest. In the future Galaxy's Crypt4GH [26] integration project will provide a more deeply integrated alternative. This ongoing project aims to implement Crypt4GH [27], a standardized encryption tool for genetic data, to encrypt data at rest in Galaxy automatically. Currently, this project does not consider memory encryption as every individual tool that works with the data must implement support for trusted compute. Alternatively, directories could be encrypted using a user key this would ensure the cached data is also encrypted.

Data Sharing

Once EGA data has been uploaded to a Galaxy instance the data and analysis can easily be shared with other users. Currently, a user could share a history containing authenticated EGA data with another user, which does not have DAC access, which is in violation with the DAA. Currently, Galaxy implements the ability to control privacy of individual datasets within a history, permitting the user to share the analysis results, without sharing the private source data. However, this remains a manual process. Therefore, we propose that in the future the permission to share DAC access datasets to another Galaxy user are validated automatically. We suggest that this validation should be dataset specific e.g., statistics, figures, and workflows derived from the history should be shareable as long as it is in compliance with the DAA.

Linking Major Repositories

In addition to the EGA other major data repositories exist which have not been linked to Galaxy yet, such as The Cancer Genome Atlas Program (TCGA) [28]. The TCGA has its own data retrieval tool, the GDC Data Transfer Tool [29], for which separate credentials within a user's Galaxy account have to be implemented. It would be more efficient for these repositories to support passports. This would greatly simplify the adoption of other major repositories in Galaxy in a GA4GH compliant way, ultimately increasing the adoption of FAIR data principles.

An alternative to linking the repositories to Galaxy, is to deploy a Galaxy instance where the data is stored. This would still require the Galaxy instance to download the data from the repository in the Galaxy instance, but the data will never leave the repository itself. However, this would require the data repository to have sufficient computing utilities in order to analyse the data with Galaxy. In the future this might be a better alternative to linking the repositories itself.

Conclusion

In this study we implemented PyEGA3 in Galaxy to retrieve data from the EGA in a GA4GH compliant manner. In addition, gene.iobio was implemented to improve variant analyses in Galaxy. These tools were validated by using B1MG data from the EGA and creating a findable analysis workflow into Galaxy. This work illustrates that gene.iobio is a major improvement compared to the current trio analysis tool in Galaxy as it creates interpretable and dynamic plots. In addition, we showed that Galaxy makes it feasible and manageable for any researcher to retrieve data from the EGA securely and analyse family trio data in a FAIR manner. Not only is this work applicable to trio analysis, it is also transferable to other omics analysis, such as genome assembly, metabolomics, metagenomics, proteomics, and transcriptomics. In conclusion, this work illustrates that Galaxy is one step closer to becoming a generalised omics platform for FAIR data analysis.

Methods

Implementation

The installation and dependencies for gene.iobio are handled by Galaxy (RRID:SCR_006281). The version of gene.iobio reported here is v4.7.1a.

Training Materials

Our workflow simplifies the data collection from the EGA and the visualization and analysis of family trios. In addition, we created a tutorial for running the workflow on Galaxy. Also, the tutorial describes in detail how to gain access to datasets on the EGA to simplify the adoption of this workflow for other data on the EGA. The tutorial is available at the Galaxy training materials website [20].

Pre-Processing

Firstly, a 'chr' prefix is added to the first column of each chromosomal site in the VCFs, to match it with the built-in reference genome from Galaxy (hg19). Secondly, the VCFs are normalized using bcftools [30]. The normalization process includes left aligning insertion or deletion and splitting multiallelic sites into biallelic records. Thirdly, the VCFs in the EGA dataset are actually genomic variant calling format (GVCFs) files. A GVCF has a record for (almost) all sites even when no variant, denoted by <NON_REF>, is recorded. In this study this information is not informative and slows down analysis. Therefore, the records with a <NON_REF> site are filtered out. Fourthly, the VCFs are merged into a single VCF based on their trio pairing using bcftools. This creates a VCF where each record also has a presence/absence column for each family pair. Finally, the variants in the merged VCF are filtered and annotated using the SnpEff tool [4] as required by GEMINI.

gene.iobio

Gene.iobio is run with the GRCh37 reference genome. The gene list is created using the phenotypes described in Additional file 1. The default search filters are used to detect the causative variant.

GEMINI

For each case GEMINI (RRID:SCR_014819) is prompted to remove low impact severity variants and to search for causative variants that match the inheritance pattern in Additional file 1.

Availability of source code and requirements

Galaxy Resources

- Galaxy Home Page: https://galaxyproject.org/
- Galaxy Tutorials: https://training.galaxyproject.org
- How to install Galaxy: https://getgalaxy.org
- How to install tools: https://galaxyproject.org/admin/tools/add-tool-from-toolshed-tutorial/
- Full administrative resources: https://docs.galaxyproject.org/
- Galaxy Help Forum: https://help.galaxyproject.org/
- Connect with the Galaxy Community on Gitter Chat: https://gitter.im/galaxyproject/Lobby/

ToolShed

- Project name: ToolShed repositories maintained by the Intergalactic Utilities Commission
- GitHub repository: https://github.com/galaxyproject/tools-iuc
- ToolShed repository: https://toolshed.g2.bx.psu.edu/
- License: MIT

PyEGA3

- Project name: pyega3 - uses the EGA REST API to download authorized datasets and files
- GitHub repository: https://github.com/galaxyproject/tools-iuc/tree/master/tools/pyega3
- ToolShed repository: https://toolshed.g2.bx.psu.edu/view/iuc/ega_download_client
- Training Manual: https://training.galaxyproject.org/training-material/topics/variant-analysis/tutorials/trio-analysis/tutorial.html
- Operating system(s): Unix (Platform independent with Docker, Singularity)
- Other requirements: Galaxy version 22.05 or higher
- License: MIT
- RRID: SCR_024654

gene.iobio

- Project name: gene.iobio - an interactive tool for variant and trio analysis
- GitHub repository: https://github.com/galaxyproject/tools-iuc/tree/master/tools/geneiobio
- ToolShed repository: https://toolshed.g2.bx.psu.edu/view/iuc/geneiobio
- Training Manual: https://gxy.io/GTN:T00320
- Operating system(s): Unix (Platform independent with Docker, Singularity)
- Other requirements: Galaxy version 22.05 or higher
- License: MIT

Availability of supporting data and materials

The data used in this study were generated by a public human WGS experiment in the Illumina Platinum initiative [31], which was made available by the HapMap project [32]. All data from this project is available at the EGA website [12]. In this study only the BAMs and VCFs with the chromosomes containing the spiked-in variants were included. These files are available under 'RD-Connect GPAP synthetic data spiked-in variant data' at Zenodo [33]. Other data further supporting this work are openly available in the GigaScience repository, GigaDB [34].

Additional Files

Additional file 1. The report describing the family trios.

Declarations

List of abbreviations

AAI: Authentication and Authorisation; BAM: Binary alignment map; B1MG: Beyond 1 Million Genomes; DAA: Data Access Agreement; DAC: Data Access Committee; EGA: European Genome-Phenome Archive; FAIR: Findable, Accessible, Interoperable, and Reusable; GA4GH: Global Alliance for Genomics and Health; GDPR: General Data Protection Regulation; GTN: Galaxy Training Network; GVCF: Genomic variant calling format; OIDC: OpenID Connect; SNP: Single nucleotide polymorphism; TCGA: The Cancer Genome Atlas Program; VCF: Variant call format.

Competing Interests

The authors declare that they have no competing interests.

Funding

This work has received funding from the EC H2020 project CINECA (grant 825775) [35] as well as the Erasmus+ programme of the European Union (Gallantries Project, grant 2020-1-NL01-KA203-064717, doi:10.13039/100001501). The data used in this project was created with the support of the RD-Connect GPAP [36], EC H2020 project EJP-RD (grant 825575) [37], EC H2020 project B1MG (grant 951724) [38], and Generalitat de Catalunya project VEIS (grant 001-P-001647) [39].

Author's Contributions

J.O., S.H., H.R., and D.S. contributed to writing the manuscript. J.O., S.H., H.R. contributed to writing the Galaxy workflow tutorial. S.H., H.R., and D.D. contributed to implementing the PyEGA3 and gene.iobio tool in Galaxy. A.P.S. supervised the project. All authors approved the manuscript.

Acknowledgements

We would like to thank the Galaxy community and in particular Wolfgang Maier, for reviewing, testing, and validating the workflow tutorial, and PyEGA3.

References

1. Inau ET, Sack J, Waltemath D, Zeleke AA. Initiatives, Concepts, and Implementation Practices of FAIR (Findable, Accessible, Interoperable, and Reusable) Data Principles in Health Data Stewardship Practice: Protocol for a Scoping Review. JMIR Research Protocols 2021;10:e22505.
2. Wilkinson MD, Dumontier M, Aalbersberg IJ, Appleton G, Axton M, Baak A, et al. The FAIR Guiding Principles for scientific data management and stewardship. Scientific Data 2016;3.
3. Nicorici D, Şatalan M, Edgren H, Kangaspeska S, Murumagi A, Kallioniemi O, et al. FusionCatcher: a tool for finding somatic fusion genes in paired-end RNA-sequencing data. bioRxiv 2014;.
4. Cingolani P, Platts A, Wang LL, Coon M, Nguyen T, Wang L, et al. A program for annotating and predicting the effects of single nucleotide polymorphisms, SnpEff. Fly 2012;6:80–92.
5. Danecek P, Bonfield JK, Liddle J, Marshall J, Ohan V, Pollard MO, et al. Twelve years of SAMtools and BCFtools. GigaScience 2021;10.
6. Krzywinski M, Schein J, Birol İ, Connors J, Gascoyne R, Horsman D, et al. Circos: An information aesthetic for comparative genomics. Genome Research 2009;19:1639–1645.
7. Jalili V, Afgan E, Gu Q, Clements D, Blankenberg D, Goecks J, et al. The Galaxy platform for accessible, reproducible and collaborative biomedical analyses: 2020 update. Nucleic Acids Research 2020;48:W395–W402.
8. Goble C, Soiland-Reyes S, Bacall F, Owen S, Williams A, Eguinoa I, et al., Implementing FAIR Digital Objects in the EOSC-Life Workflow Collaboratory.; 2021. https://zenodo.org/record/4605654, accessed: 2022-03-21.
9. Galaxy Training Network Stats;. https://training.galaxyproject.org/stats, accessed: 2022-10-27.
10. Rasche H, Hiltemann S. Galactic Circos: User-friendly Circos plots within the Galaxy platform. GigaScience 2020;9.
11. Lappalainen I, Almeida-King J, Kumanduri V, Senf A, Spalding JD, ur Rehman S, et al. The European Genome-phenome Archive of human data consented for biomedical research. Nature Genetics 2015;47(7):692–695.
12. Freeberg MA, Fromont LA, D'Altri T, Romero AF, Ciges J, Jene A, et al. The European Genome-phenome Archive in 2021. Nucleic Acids Research 2021;50:D980–D987.
13. Beyond 1 Million Genomes;. https://b1mg-project.eu/, accessed: 2022-06-30.
14. Sera TD, Velinder M, Ward A, Qiao Y, Georges S, Miller C, et al. Gene.iobio: an interactive web tool for versatile, clinically-driven variant interrogation and prioritization. Scientific Reports 2021;11.
15. Hiltemann S, Rasche H, Gladman S, Hotz HR, Larivière D, Blankenberg D, et al. Galaxy Training: A powerful framework for teaching! PLOS Computational Biology 2023;19(1):1–18.
16. Galaxy Vault;. https://docs.galaxyproject.org/en/master/admin/special_topics/vault.html, accessed: 2023-09-25.
17. Paila U, Chapman BA, Kirchner R, Quinlan AR. GEMINI: Integrative Exploration of Genetic Variation and Genome Annotations. PLOS Computational Biology 2013;9:1–8.
18. Rare Disease Synthetic Dataset;. https://ega-archive.org/datasets/EGAD00001008392, accessed: 2022-10-06.
19. Galaxy Training Network Stats;. https://training.galaxyproject.org/, accessed: 2022-10-27.
20. Trio Analysis using Synthetic Datasets from RD-Connect GPAP;. https://training.galaxyproject.org/training-material/topics/variant-analysis/tutorials/trio-analysis/tutorial.html, accessed: 2022-09-23.
21. Trio Analysis;. https://workflowhub.eu/workflows/363, accessed: 2023-09-05.
22. Yang H, Robinson PN, Wang K. Phenolyzer: phenotype-based prioritization of candidate genes for human diseases. Nature Methods 2015;12:841–843.
23. Voisin C, Linden M, Dyke SOM, Bowers SR, Alper P, Barkley MP, et al. GA4GH Passport standard for digital identity and access permissions. Cell Genomics 2021;1(2):100030.
24. General Data Protection Regulation;. https://gdpr-info.eu/, accessed: 2023-09-13.
25. Using server-side encryption with customer-provided keys (SSE-C);. https://docs.aws.amazon.com/AmazonS3/latest/userguide/ServerSideEncryptionCustomerKeys.html#specifying-s3-c-encryption, accessed: 2023-09-29.
26. Galaxy Crypth4GH Recryptor Service;. https://github.com/elixir-europe/GalaxySensitiveData-ELIXIR_IS, accessed: 2023-09-27.
27. Senf A, Davies R, Haziza F, Marshall J, Troncoso-Pastoriza J, Hofmann O, et al. Crypt4GH: a file format standard enabling native access to encrypted data. Bioinformatics 2021;37:2753–2754.
28. Program TCGA;. https://www.cancer.gov/ccg/research/genome-sequencing/tcga, accessed: 2023-09-13.
29. cancer Institute: Genomic Data Commons N;. https://github.com/NCI-GDC/gdc-client, accessed: 2023-09-13.
30. Danecek P, Bonfield JK, Liddle J, Marshall J, Ohan V, Pollard MO, et al. Twelve years of SAMtools and BCFtools. GigaScience 2021;10.
31. Eberle MA, Fritzilas E, Krusche P, Källberg M, Moore BL, Bekritsky MA, et al. A reference data set of 5.4 million phased human variants validated by genetic inheritance from sequencing a three-generation 17-member pedigree. Genome Research 2016;27:157–164.
32. HapMap Project;. www.genome.gov/10001688/international-hapmap-project, accessed: 2022-11-02.
33. Ouwerkerk J, Zenodo: RD-Connect GPAP synthetic data; 2022. https://doi.org/10.5281/zenodo.7273767.
34. Ouwerkerk J, Rasche H, Spalding JD, Hiltemann S, Stubbs AP, Supporting data for "FAIR Data Retrieval for Sensitive Clinical Research Data in Galaxy" GigaScience Database; 2023. http://dx.doi.org/10.5524/102472.
35. CINECA Project;. https://cordis.europa.eu/project/id/825775, accessed: 2022-11-02.
36. RD-Connect GPAP Project;. https://platform.rd-connect.eu/, accessed: 2022-11-02.
37. EJP-RD Project;. https://cordis.europa.eu/project/id/825575, accessed: 2022-11-02.
38. B1MG Project;. https://cordis.europa.eu/project/id/951724, accessed: 2022-11-02.
39. VEIS Project;. http://www.gcatbiobank.org/investigadors/projects/25/veis-european-project-valuation-of-the-european-archive-
accessed: 2022-11-02.
