# Supplementary material for: FAIR data retrieval for sensitive clinical research data in Galaxy
Source: Gigascience. 2024 Jan 27;13:giad099. doi: 10.1093/gigascience/giad099 (PMC10821763; doi:10.1093/gigascience/giad099)
Supplement: giad099_Response_to_Reviewer_Comments_Original_Submission [file giad099_response_to_reviewer_comments_original_submission.pdf]

Author's Response To Reviewer Comments

Dear editors,

Thank you for your dedicated time, comments and knowledge for our manuscript. We have updated the manuscript to address your comments. All the specific changes and line numbers can be found in the additional revision1.pdf file.
We hope that the manuscript is now ready for publication in the GigaScience journal.

Kind regards,

Jasper Ouwerkerk (MSc)
PhD candidate
Department of Pathology & Clinical Bioinformatics

On behalf of all authors.

Editor:
1. Please register any new software application in the bio.tools and SciCrunch.org databases to receive RRID (Research Resource Identification Initiative ID) and biotoolsID identifiers, and include these in your manuscript. Computational workflows should be registered in workflowhub.eu and the DOIs cited in the relevant places in the manuscript. These will facilitate tracking, reproducibility and re-use of your tool.

We have added the DOI of the workflow on workflowhub.eu to the citations.

Reviewer #1:
Main comments:
1. The authors explore the limitations of PyEGA3 on Galaxy regarding data retrieval; however, they fail to discuss data management after retrieval. With PyEGA3, files are transferred over secure HTTPS connections but received unencrypted. It follows that genomic data are stored as unencrypted datasets on the Galaxy account of the user once retrieved from EGA. Also, any output file resulting from their processing is stored as unencrypted data by Galaxy. This means PyEGA3 should not be deployed on any Galaxy instance over which the institution of the user has no direct control. In fact, the standard EGA Data Access Agreement (DAA) states that "The User Institution agrees to preserve, at all times, the confidentiality of these Data." this cannot be achieved unless it has complete control over data access. Furthermore, the DAA states, "The User Institution agrees only to transfer or disclose these Data, in whole or part, or any material derived from these Data, to the Authorised Personnel. Should the User Institution wish to share these Data with an External Collaborator, the External Collaborator must complete a separate application for access to these Data". In practice, those provisions forbid the use of public servers for the analysis of EGA datasets. However, many Galaxy instances are public (e.g. usegalaxy.org, usegalaxy.eu), and potential users of a tool like PyEGA3 may not be aware of this issue.

We thank the reviewer for their suggestions. We agree that data management is very relevant, especially for data from the EGA, and we acknowledge that our manuscript did not clearly raise awareness for this issue. Therefore, we have added a “Data Management” subsection in the “Limitations and Future Work” section. In the first paragraph of the “Data Management” section (line 209-216) we highlight that data is stored unencrypted on Galaxy instances and that uploading confidential data from the EGA to a public Galaxy instance would violate the DAA. In addition, we have added a warning to the PyEGA tool in Galaxy to raise the user's awareness of the DAA, stating:
"Data is stored unencrypted on the user's Galaxy account. Confidential data from the EGA could be left unprotected when uploaded to a public Galaxy server. Make sure to read the EGA Data Access Agreement (DAA) before uploading any data to Galaxy from the EGA. If this applies to you, we recommend you follow https://github.com/elixir-europe/GalaxySensitiveData-ELIXIR_IS for updates on encrypted data at rest."

2. The authors should discuss how and when their approach is usable for processing human genetic data in

compliance with EGA guidelines and GDPR. Considering the above, a possible solution would be to keep the data encrypted until use and to protect the output files similarly. Confidential computing during processing would also be desirable but out of scope for the present work. An alternative would be to process data on private Galaxy instances owned or controlled by the user's institution rather than on public ones.

We acknowledge that our manuscript did not sufficiently explain how our approach could be used in compliance with the EGA and GDPR guidelines when using sensitive human genetic data. Therefore, we added a second paragraph to the “Data Management” subsection (line 217-231) explaining that our approach is only in compliance with the EGA and GDPR guidelines when the data is uploaded to a private Galaxy managed by the user's institution. In addition, we describe an ongoing project to encrypt data in Galaxy using Crypt4GH, possible improvements, and alternatives to this project.

3. In the results section, the authors write: "Authentication of the user [to EGA] is either done by password and username or Authentication and Authorization Infrastructure (AAI) tokens." When username/password authentication is used, it is unclear how Galaxy manages those sensitive data. Are they stored somewhere? In this case, how are they protected?

To clarify, we have added an explanation to the manuscript (line 72-78) describing where the username and password and AAI tokens are stored and how this data is protected.

Minor comments:
1. I find the title misleading since the tool presented in the article is limited to data retrieval from EGA. So it is about "clinical research data" that has already been deposited in that repository and thus cannot reasonably support the day-to-day work of clinical data analysis in a healthcare setting.

As we want to avoid any potential misleading of the reader, we decided to revise the title and changed it to: “FAIR Data Retrieval for Sensitive Clinical Research Data in Galaxy”.
2. Avoid using expressions like "Galaxy is a great platform" and stick to more formal and precise writing.

We have checked the manuscript and formalized expressions where needed.

Reviewer #2:
Main comments:
1. One advantage of Galaxy is the ability to share complete analysis Histories. Is it possible to share a history that uses authenticated data from EGA using this new PyEGA3 service? Is sharing of analyses only limited to workflows? Perhaps it would be possible to share a History that contains controlled raw data if the other user has appropriate authentication credentials. Some clarification on how sharing can occur with a tool such as this would be helpful.

We thank the reviewer for their suggestions and acknowledge that we did not touch upon data sharing with regards to EGA data. Therefore, we added a subsection “Data Sharing” under the “Limitations & Future Work” section (line 233-245). Here we acknowledge that currently a Galaxy user could share a dataset or history with EGA data to another Galaxy user without DAC access, which would violate the DAA. We propose, in the paragraph, that in the future data sharing in Galaxy should be validated with the DAA in mind for EGA data.

2. The authors have designed this service to access EGA data, which is a major repository. Could such a tool also be modified to access data from other controlled data sources - for example the US NIH repositories such as TCGA? Would implementing the GA4GH standards as discussed in the manuscript enable this broader usage?

We agree that it would be very relevant to link other major repositories such as the TCGA to Galaxy. Linking other major repositories would definitely be accelerated if the GA4GH standards would have been

implemented by such repositories. Therefore, we added a subsection “Linking Major Repositories” in the “Limitations & Future Work” section discussing this issue in the first paragraph (line 247-255).

3. It looks as though PyEGA3 works by downloading data from the EGA repository. Is there potential in the future to utilize PyEGA3 in a cloud-based Galaxy instance that could be launched where the data itself "lives" rather than requiring data download and upload? This may be a future possibility but some mention may be helpful.

This would indeed be a good future solution to prevent data transfer from one repository to another. We have discussed this solution in the second paragraph of the “Linking Major Repositories” subsection (line 256-263).

Minor comments:
1. A bit more description in the manuscript about the details of "Trio" analysis could be helpful for those readers not familiar with this type of genomic analysis.

We agree that we haven't fully explained the concept of trio-analysis in our manuscript. We have added a more detailed description of trio analysis in the last paragraph of the background section (line 55-59).

2. The gene.iobio tool has been published by others as a standalone software. This could be made a bit more clear when it is first mentioned in the manuscript.

We agree that initially it might not have been very clear to the reader whether the gene.iobio tool was a tool on its own. This has been clarified in the last paragraph of the background section (line 59-61).
