# Supplementary material for: FAIR data retrieval for sensitive clinical research data in Galaxy
Source: Gigascience. 2024 Jan 27;13:giad099. doi: 10.1093/gigascience/giad099 (PMC10821763; doi:10.1093/gigascience/giad099)
Supplement: giad099_Response_to_Reviewer_Comments_Revision_1 [file giad099_response_to_reviewer_comments_revision_1.pdf]

Author's Response To Reviewer Comments

Dear editors,

Thank you for reviewing and provisionally accepting our manuscript. We have updated the manuscript to address the suggested edits. We hope the manuscript is now ready for publication in the GigaScience journal.

Kind regards,

Jasper Ouwerkerk (MSc)
PhD candidate
Department of Pathology & Clinical Bioinformatics

On behalf of all authors.

Editor:

Your manuscript "FAIR Data Retrieval for Sensitive Clinical Research Data in Galaxy" (GIGA-D-23-00177R1) has been assessed by our reviewers. Based on these reports, and my own assessment as Editor, I am pleased to inform you that it is potentially acceptable for publication in GigaScience, once you have carried out some essential formatting revisions suggested by our Editor.
Please see the attached marked up PDF with comments. Note the GigaDB DOI citation needs to be added to the paper and also the References - this has been marked up.
We have updated our manuscript according to your comments. We added the RRIDs where needed and the GigaDB citation of our supporting data to the manuscript.
