# Supplementary material for: FAIR data retrieval for sensitive clinical research data in Galaxy
Source: Gigascience. 2024 Jan 27;13:giad099. doi: 10.1093/gigascience/giad099 (PMC10821763; doi:10.1093/gigascience/giad099)
Supplement: giad099_Reviewer_1_Report_Original_Submission — Federico Zambelli -- 7/24/2023 Reviewed [file giad099_reviewer_1_report_original_submission.pdf]

Reviewer Report

Title: FAIR Data Retrieval for Sensitive Clinical Research Data in Galaxy

Version: Original Submission **Date:** 7/24/2023

Reviewer name: Federico Zambelli

Reviewer Comments to Author:

Ouwerkerk et al. present an implementation of the PyEGA3 tool for downloading and using EGA datasets within the popular Galaxy workflow manager for bioinformatics. Besides, they also port to Galaxy the Gene.iobio tool for variant analysis and prioritization and develop a workflow for trio analysis. Finally, the authors showcase the use of the two tools with a synthetic dataset use case.Efforts towards developing tools for making the processing of genomic data from clinical research more accessible are welcome and much needed. Easy retrieving data from EGA on a user-friendly platform like Galaxy can be considered a step in the right direction. However, I find a few issues that the authors have partially overlooked and should be discussed in the article to convey better information to the reader.In particular:- The authors explore the limitations of PyEGA3 on Galaxy regarding data retrieval; however, they fail to discuss data management after retrieval. With PyEGA3, files are transferred over secure HTTPS connections but received unencrypted. It follows that genomic data are stored as unencrypted datasets on the Galaxy account of the user once retrieved from EGA. Also, any output file resulting from their processing is stored as unencrypted data by Galaxy. This means PyEGA3 should not be deployed on any Galaxy instance over which the institution of the user has no direct control. In fact, the standard EGA Data Access Agreement (DAA) states that "The User Institution agrees to preserve, at all times, the confidentiality of these Data." this cannot be achieved unless it has complete control over data access. Furthermore, the DAA states, "The User Institution agrees only to transfer or disclose these Data, in whole or part, or any material derived from these Data, to the Authorised Personnel. Should the User Institution wish to share these Data with an External Collaborator, the External Collaborator must complete a separate application for access to these Data". In practice, those provisions forbid the use of public servers for the analysis of EGA datasets. However, many Galaxy instances are public (e.g. usegalaxy.org, usegalaxy.eu), and potential users of a tool like PyEGA3 may not be aware of this issue.- The authors should discuss how and when their approach is usable for processing human genetic data in compliance with EGA guidelines and GDPR. Considering the above, a possible solution would be to keep the data encrypted until use and to protect the output files similarly. Confidential computing during processing would also be desirable but out of scope for the present work. An alternative would be to process data on private Galaxy instances owned or controlled by the user's institution rather than on public ones.- In the results section, the authors write: "Authentication of the user [to EGA] is either done by password and username or Authentication and Authorization Infrastructure (AAI) tokens." When username/password authentication is used, it is unclear how Galaxy manages those sensitive data. Are they stored somewhere? In this case, how are they protected?Minor:- I find the title misleading since the tool presented in the article is limited to data retrieval from EGA. So it is about "clinical research data" that has already been deposited in that repository and thus cannot reasonably support the day-to-

day work of clinical data analysis in a healthcare setting.- Avoid using expressions like "Galaxy is a great platform" and stick to more formal and precise writing.

Level of Interest

Please indicate how interesting you found the manuscript: Choose an item.

Quality of Written English

Please indicate the quality of language in the manuscript: Choose an item.

Declaration of Competing Interests

Please complete a declaration of competing interests, considering the following questions:

- Have you in the past five years received reimbursements, fees, funding, or salary from an organisation that may in any way gain or lose financially from the publication of this manuscript, either now or in the future?
- Do you hold any stocks or shares in an organisation that may in any way gain or lose financially from the publication of this manuscript, either now or in the future?
- Do you hold or are you currently applying for any patents relating to the content of the manuscript?
- Have you received reimbursements, fees, funding, or salary from an organization that holds or has applied for patents relating to the content of the manuscript?
- Do you have any other financial competing interests?
- Do you have any non-financial competing interests in relation to this paper?

If you can answer no to all of the above, write 'I declare that I have no competing interests' below. If your reply is yes to any, please give details below.

I declare that I have no competing interests.

I agree to the open peer review policy of the journal. I understand that my name will be included on my report to the authors and, if the manuscript is accepted for publication, my named report including any attachments I upload will be posted on the website along with the authors' responses. I agree for my report to be made available under an Open Access Creative Commons CC-BY license (http://creativecommons.org/licenses/by/4.0/). I understand that any comments which I do not wish to be included in my named report can be included as confidential comments to the editors, which will not be published.

Choose an item.

To further support our reviewers, we have joined with Publons, where you can gain additional credit to further highlight your hard work (see: https://publons.com/journal/530/gigascience). On publication of this paper, your review will be automatically added to Publons, you can then choose whether or not to claim your Publons credit. I understand this statement.

Yes Choose an item.
