# Supplementary material for: FAIR data retrieval for sensitive clinical research data in Galaxy
Source: Gigascience. 2024 Jan 27;13:giad099. doi: 10.1093/gigascience/giad099 (PMC10821763; doi:10.1093/gigascience/giad099)
Supplement: giad099_Reviewer_2_Report_Original_Submission — Timothy Griffin -- 9/3/2023 Reviewed [file giad099_reviewer_2_report_original_submission.pdf]

Reviewer Report

Title: FAIR Data Retrieval for Sensitive Clinical Research Data in Galaxy

Version: Original Submission **Date:** 9/3/2023

Reviewer name: Timothy Griffin

Reviewer Comments to Author:

The authors present a a new Galaxy service called PyEGA3 built to access authenticated, controlled data from the European Genome-Phenome Archive (EGA) for reanalysis and interrogation using tools available in Galaxy. They also implement the tool gene.iobio tool in order to characterize and visualize annotatied data accessed using the PyEGA3 tool. In order to realize the potential of repositories such as EGA, there is a need for services such as PyEGA3 to enable accesss to controlled data and transfer to platforms such as Galaxy where further analysis can be carried out to extend the discoveries possible from these rich datasets. The authors describe the service clearly, and have made the PyEGA3 and gene.iobio tools available openly in both Github and the Galaxy Tool Shed. They have also developed online training materials that are openly and freely available in the Galaxy Training Network, which should empower others to utilize this tool. This is sound work, however there are some comments that the authors could answer in a revision in order to strengthen the manuscript.Main comments1. One advantage of Galaxy is the ability to share complete analysis Histories. Is it possible to share a history that uses authenticated data from EGA using this new PyEGA3 service? Is sharing of analyses only limited to workflows? Perhaps it would be possible to share a History that contains controlled raw data if the other user has appropriate authentication credentials. Some clarification on how sharing can occur with a tool such as this would be helpful.2. The authors have designed this service to access EGA data, which is a major repository. Could such a tool also be modified to access data from other controlled data sources - for example the US NIH repositories such as TCGA? Would implementing the GA4GH standards as discussed in the maniuscript enable this broader usage?3. It looks as though PyEGA3 works by downloading data from the EGA repository. Is there potential in the future to utilize PyEGA3 in a cloud-based Galaxy instance that could be launched where the data itself"lives" rather than requiring data download and upload? This may be a future possibility but some mention may be helpful.Minor comments1. A bit more description in the manuscript about the details of "Trio" analysis could be helpful for those readers not familiar with this type of genomic analysis.2. The gene.iobio tool has been published by others as a standalone software. This could be made a bit more clear when it is first mentioned in the manuscript.

Level of Interest

Please indicate how interesting you found the manuscript: Choose an item.

Quality of Written English

Please indicate the quality of language in the manuscript: Choose an item.

Declaration of Competing Interests

Please complete a declaration of competing interests, considering the following questions:

- Have you in the past five years received reimbursements, fees, funding, or salary from an organisation that may in any way gain or lose financially from the publication of this manuscript, either now or in the future?
- Do you hold any stocks or shares in an organisation that may in any way gain or lose financially from the publication of this manuscript, either now or in the future?
- Do you hold or are you currently applying for any patents relating to the content of the manuscript?
- Have you received reimbursements, fees, funding, or salary from an organization that holds or has applied for patents relating to the content of the manuscript?
- Do you have any other financial competing interests?
- Do you have any non-financial competing interests in relation to this paper?

If you can answer no to all of the above, write 'I declare that I have no competing interests' below. If your reply is yes to any, please give details below.

I declare that I have no competing interests

I agree to the open peer review policy of the journal. I understand that my name will be included on my report to the authors and, if the manuscript is accepted for publication, my named report including any attachments I upload will be posted on the website along with the authors' responses. I agree for my report to be made available under an Open Access Creative Commons CC-BY license (http://creativecommons.org/licenses/by/4.0/). I understand that any comments which I do not wish to be included in my named report can be included as confidential comments to the editors, which will not be published.

Choose an item.

To further support our reviewers, we have joined with Publons, where you can gain additional credit to further highlight your hard work (see: https://publons.com/journal/530/gigascience). On publication of this paper, your review will be automatically added to Publons, you can then choose whether or not to claim your Publons credit. I understand this statement.

Yes Choose an item.
